# Genetic diversities and phylogenetic analyses of three Chinese main ethnic groups in southwest China: A Y-Chromosomal STR study

**DOI:** 10.1038/s41598-018-33751-x

**Published:** 2018-10-18

**Authors:** Pengyu Chen, Guanglin He, Xing Zou, Xin Zhang, Jida Li, Zhisong Wang, Hongyan Gao, Li Luo, Zhongqing Zhang, Jian Yu, Yanyan Han

**Affiliations:** 1 Center of Forensic Expertise, Affiliated Hospital of Zunyi Medical University, Zunyi, Guizhou China; 2 Department of Forensic genetics, School of Forensic Medicine, Zunyi Medical University, Zunyi, Guizhou China; 3 Institute of Forensic Medicine, West China School of Basic Medical Sciences & Forensic Medicine, Sichuan University, Chengdu, Sichuan China; 4 Department of Forensic Medicine, College of Basic Medicine, Chongqing Medical University, Chongqing, China; 5 People’s Hospital of Wuxi County, Chongqing, China; 6 School of Public Health, Zunyi Medical University, Zunyi, Guizhou China

## Abstract

Short tandem repeats (STRs) located on the Y chromosome with the properties of male-specific inheritance and haploidy are widely used in forensics to analyze paternal genealogies and match male trace donors to evidence. Besides, Y-chromosomal haplotypes play an important role in providing breathtaking insights into population genetic history. However, the genetic diversity and forensic characteristics of Y-STRs in Guizhou main ethnic groups (Hans, Miaos and Bouyeis) remain uncharacterized. Here, we obtained Y-chromosomal 23-marker haplotypes in three Guizhou populations and submitted the first batch of Y-STR haplotype data to the YHRD. The HD in the aforementioned three populations are 0.99990, 0.99983, and 0.99979, respectively, and DC values are 0.9902, 0.9908, and 0.97959, respectively. Subsequently, genetic differentiation between our newly studied populations and reference groups along ethnic/administrative divisions, as well as national/continental boundaries were investigated via AMOVA, MDS, and phylogenetic relationship reconstruction. Significant genetic differentiations from our subjects and other groups are identified in ethnically, linguistically and geographically diverse populations, including most prominently Tibetans and Uyghurs among 30 mainland Chinese populations, Taiwanese groups and others among 58 Asian populations, as well as African groups and others among 89 worldwide populations. Qiannan Bouyei has a close genetic relationship with Guangxi Zhuang, and Zunyi Han and Qiandongnan Miao have close genetic affinity with Hunan Han and Guizhou Shui, respectively. Collectively, this new-generation Y-STR amplification system can be used as a supplementary tool in forensic identification and male parentage testing and even pedigree search.

## Introduction

Recently, advances of the whole Y chromosome high-coverage sequencing have facilitated the clear understanding of the genetic variations of this unilinearly transmitted genome segment and revolutionized the insights and prospects of interdisciplinary researches^1–6^. Human Y chromosome variations, with the properties of male specificity, haploidy and escaping from crossing-over, play an important role in the studies of anthropology, genealogy, as well as population and forensic genetics. Poznik *et al*. found over 65,000 Y-chromosomal genetic variants (single nucleotide polymorphisms, SNPs; short tandem repeats, STRs; insertion/deletions, InDels; copy number variants, CNV; and multiple nucleotide variants) via massive parallel sequencing 1244 individuals from 26 diverse populations^7^. The better understanding of the human Y-chromosome variations has driven its unprecedented process in the forensic applications^8,9^. Y-STRs, also known as microsatellites located on the human Y chromosome, are tandemly repeated short (2–6 bp) DNA sequences^3–5^. Thomas Willems *et al*. have investigated the mutation rates, forensic characteristics and potential applications of 4,500 Y-STRs on the basis of population sequence data^1^. Ballantyne *et al*. found 13 most mutable Y-STRs extracting from 186 markers after analyzing the mutation rates covering 352,999 meiotic transfers in forensic male lineage identification^10^. These previous contributions have prompted the widespread use of the non-recombining Y-chromosomal microsatellites in the forensic science: inferring biological sex of perpetrators, inferring paternal bio-geographic ancestry, characterizing paternal lineages of unknown crime scene male trace donors, predicting a man’s surname, paternal and complex kinship identification, as well as familial searching^1,9,11–17^. The PowerPlex^®^ Y23 System, developed and released in 2013 by the Promega and co-amplifying 23 Y-STRs (17 typical Y-STRs included in the Y Filer kit, four highly discriminating Y-STRs and two rapidly mutable Y-STRs), plays an important role in the research of population genetic, forensic genetics, human evolution and molecular anthropology^18–20^.

China, as a multi-ethnic country which consists of Han Chinese populations and 55 officially recognized minorities, has been the research hotspot in the genetic study to explore and elucidate the population substructures and the origin of these ethnic groups^21–23^. While massive researches have concentrated on the genetic polymorphisms based on autosomal-STRs^24–27^, X-Chromosomal-STRs^28–30^ and mitochondrial genome genetic markers^31^ in distinct Chinese populations. Guizhou province, located in the southwestern part of China with a population over 34 million, is one of China’s most demographically diverse province. The first three largest populations are Han (62%), Miao (12%) and Bouyei (8%). Han ethnic group, with a population over 1.2 billion, is the world’s largest ethnic group and widely distributed in the East Asia, Southeast Asia (76% of Singapore and 23% of Malaysia) and others. According to the historical materials, Han Chinese population was believed to be decedents of confederation of Huaxia tribes which residing along the Yellow River in the north of China^21,31,32^. Previous studies hold the opinion that a significant difference has existed between the northern Hans and southern Hans, but, others found just continuous genetic North–South gradient in the Chinese Han population^21,23,31–34^. Besides, as we all known that, the observed population substructure could be complicated by many factors, such as the number and kinds of genetic markers, the geographic origins or coverage of studied populations and reference populations^23,33,34^. Miao has a population of approximately 12 million and lives primarily in the southern Chinese mountains, whose language family belongs to the Hmong–Mien language family. The Bouyei, as the eleventh largest ethnicity with approximately 2.5 million population, live in semi-tropical, high-altitude forests of the southwest China. Previous historical evidence suggested that Bouyei is the decedents of the oldest Tai people and Bouyei language is very close to the Zhuang language and belongs to Tai-Kadai the language family.

However, little is known about the diversity of the aforementioned Y-STRs in Guizhou populations. Y chromosome haplotype reference database (YHRD) is contributed to provide a large and high quality Y-STR haplotype data from distinct genetic populations across the world for forensic and population genetic applications and researches^12^. As yet, Y chromosome variation data in Guizhou populations, especially for Chinese Bouyei and Guizhou Miao, remains blank.

In the continuation of our previous studies^35–37^, we investigated the Y-chromosomal STR haplotype distributions in three Guizhou ethnicities in the southwestern China using the PowerPlex Y23 amplification system (Promega, Madison, USA)^20^ and submitted the first batch of Y-STR haplotype data of Zunyi Han (YA004233), Qiandongnan Miao (YA004235) and Qiannan Bouyei (YA004236) for forensic, genealogical, and ethnic evolutionary researches. Additionally, we combined our new datasets with previously published reference populations^19,23,35–44^ defined by ethnic and administrative boundaries, as well as national and continental geographic divisions (eight Han Chinese populations, nineteen Chinese minority ethnicities, fifty-eight Asian populations, seven Meta-populations, and eighty-nine worldwide populations). With this unprecedented dataset, we aimed to comprehensively characterize genetic relationships and reconstruct phylogenetic history.

## Results

### Y-chromosomal genetic diversity in Guizhou Bouyei, Han and Miao

We genotyped a total of 308 Guizhou individuals successfully and provided the first Y-chromosome haplotype data of three studied populations, as showed in Supplementary Tables 1–3 Forensic parameters, including allele frequencies and gene diversity (GD), are listed in Supplementary Tables 4–6 For the Bouyei population, 96 different haplotypes are observed from 98 individuals. Among them, 94 are unique and 2 are duplicates (Supplementary Table S1). A total of 160 alleles with the corresponding allele frequencies varying from 0.0030 to 0.7857 are observed (Supplementary Table S4). The gene diversity (GD) spans from 0.3545 at the locus of DYS391 to 0.9520 at the locus of DYS385a/b, followed by DYS458 (0.8550) with the average and standard deviation are 0.6806 ± 0.1369. GD values are larger than 0.5 with the exceptions of DYS391 (0.3545) and DYS437 (0.4639). The haplotype frequencies (HF) vary from 0.0102 to 0.0204 and the random match probability (RMP) is 0.0106. The overall haplotype diversity (HD) of Guizhou Bouyei is 0.99979 and the discrimination capacity (DC) is 0.97959.

After statistical analysis of 102 Guizhou Han individuals, 101 different haplotypes in total are identified consisting of 100 singletons (99.01%) and one haplotype shared by two individuals (0.99%) (Supplementary Table S2), with the haplotype frequencies vary from 0.0098 to 0.0196. The HD is 0.99990. The values of RMP and DC are 0.009996 and 0.9902, respectively. The HD is 0.99990 in the Guizhou Han Chinese population. Additionally, two microvariants namely 13.2 and 17.2 at the locus of DYS385 were screened. As shown in Supplementary Table S5, a total of 125 alleles are observed in 21 single-copy Y-STR loci in 102 individuals with the allele frequencies range from 0.0029 to 0.7451, and 38 different allele combinations (haplotype comprising two Y-STR loci) and 15 alleles are found in the multi-copy locus of DYS385a/b with the haplotype frequencies vary from 0.0098 to 0.0784. The GD varies from 0.4015 (DYS438) to 0.9654 (DYS385a/b). All studied loci get GD values higher than 0.5 except for DYS438 (0.4015), DYS437 (0.4286), DYS391 (0.4612).

In the Miao population, the allele frequencies and GD values are presented in Supplementary Table S6. We detected a total of 115 alleles in the 21 single-copy loci with allele frequencies span from 0.0029 to 0.8532. And 35 haplotype combinations were showed (15 alleles) with the haplotype frequencies vary from 0.0092 to 0.1468 in the locus of DYS385a/b. The GD varies from 0.2615 (DYS391) to 0.9468 (DYS385a/b). Except for DYS391 (0.2615), DYS438 (0.3140), DYS437 (0.4622), DYS456 (0.4817), other studied loci have the GD values over 0.5. Supplementary Table S3 lists the haplotype information and 108 haplotypes observed. 106 haplotypes are unique (98.12%) and one is found in two individuals (1.85%) with the haplotype frequencies span from 0.0092 to 0.0183. The values of RMP, DC, and HD are 0.0093, 0.9908, and 0.99983, respectively.

### Genetic differentiation along mainland Chinese administrative and ethnic divisions

**Figure 1:**
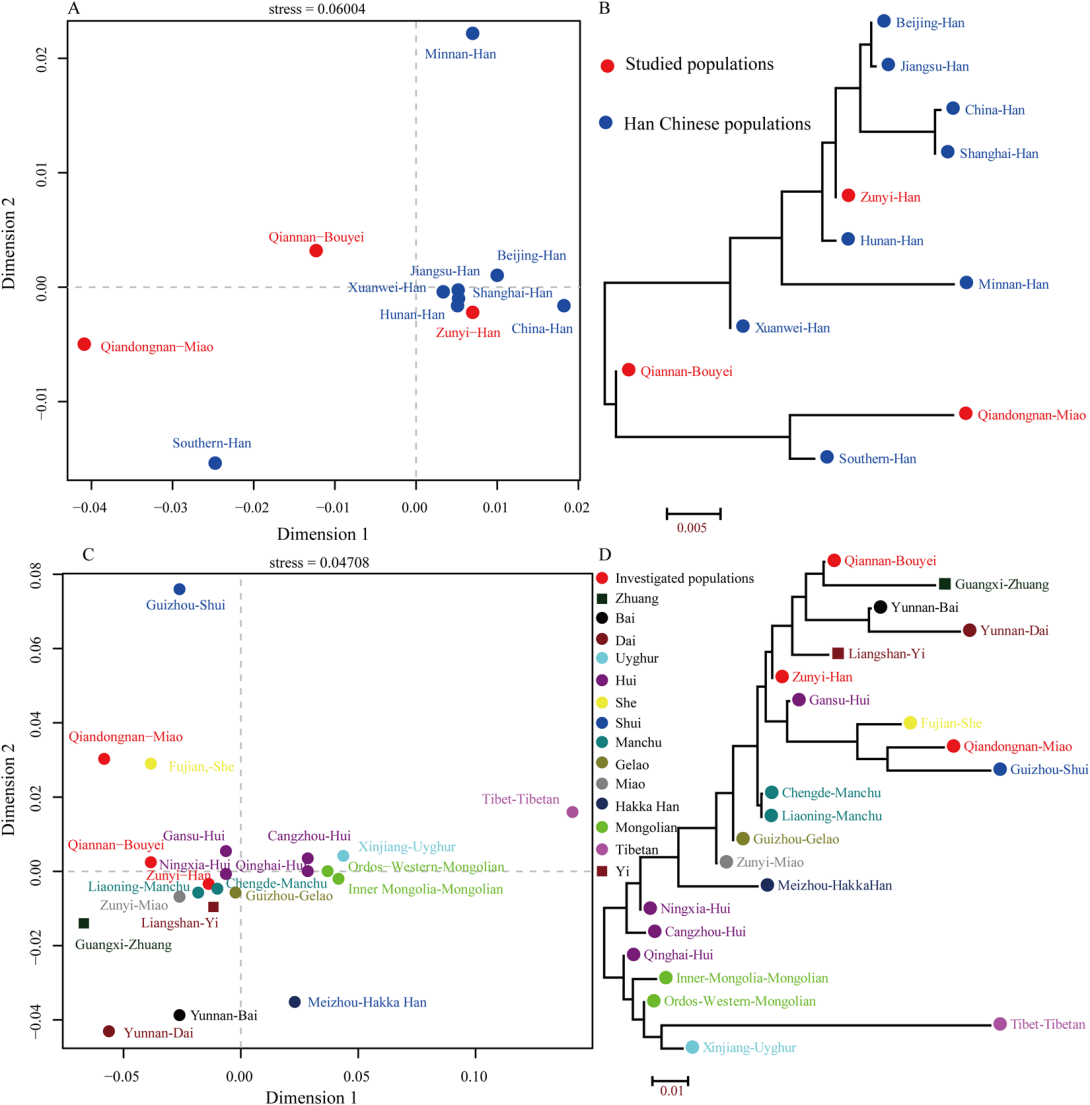
Genetic relationships between three studied populations and reference populations defined by ethnic origin and administrative divisions. (**A**) Multidimensional scaling plots show the genetic correlation between our subjects and eight Han Chinese populations; (**B**) Phylogenetic relationship between our targets and eight Han populations; (**C**) Two-dimensional scaling plots show the genetic differentiation between studied population and 19 Chinese minority ethnicities; (**D**) The Neighbor-Joining tree was constructed based on Rst genetic distance matrix among 22 populations.

3589 Y-STR haplotypes consisting of 23 markers from 11 Chinese populations^19,23,35,43^ are used to investigate the degree of differentiation between our studied three subjects and other 8 Han Chinese populations (including Minnan Han, Beijing Han, Jiangsu Han, Xuanwei Han, Shanghai Han, Hunan Han, China Han and Southern Han) via analysis of molecular variance (AMOVA), Multidimensional scaling plots (MDS) and phylogenetic relationship reconstruction. Supplementary Table S7 lists the Rst values among 11 groups and shows that the largest genetic distance is observed between Qiandongnan Miao and Shanghai Han (Rst = 0.0777). Population substructure based on pairwise genetic distance matrix is shown in Fig. 1A. Two investigated minorities (Qiandongnan Miao and Qiannan Bouyei) and two previously investigated Minnan Han and Southern Han are isolated from other seven Han Chinese populations which are located at the corner of MDS. Other Han Chinese populations (including newly genotyped Zunyi Han) keep a strong genetic affinity with each other and group together. Phylogenetic relationships among studied populations and reference Han Chinese populations distributed in different administrative divisions are shown in Fig. 1B. Two branches are observed: one consists of Qiannan Bouyei, Qiandongnan Miao and Southern Han, the other was formed by the remaining populations. Zunyi Han is subsequently clustered with Hunan Han and Minnan Han. Qiandongnan Miao is first clustered with Southern Han and then with Qiannan Bouyei. Previous genetic studies have demonstrated that a significant genetic distinction or genetic gradient between the southern Han and northern Han is observed^21,23,31–34,45–47^. However, this study based on 23 Y-STR haplotype data fails to reveal this genetic phenomenon which is explicable by the few populations from north China.

To explore the genetic homogeneity and heterogeneity among newly investigated populations and Chinese minority ethnic groups^19,23,35–41^, 4620 Y-STR haplotypes from 22 populations are employed to calculate the pairwise Rst values. As shown in Supplementary Table S8, the largest genetic distance is observed between Yunnan Dai and Tibet Tibetan (Rst = 0.2344). Zunyi Han, Qiandongnan Miao and Qiannan Bouyei have the close genetic relationships with Chengde Manchu (0.0030), Qiannan Bouyei (0.0171) and Zunyi Han (0.0153), respectively. And Tibetan is the most distantly related to our studied Zunyi Han, Qiandongnan Miao and Qiannan Bouyei (0.1556, 0.2263, and 0.1747, respectively). MDS results reveal substantial genetic distances among Chinese ethnicities, especially in between Guizhou Shui, Tibet Tibetan, Yunnan Dai, Yunnan Bai, Meizhou Hakka Han, Guangxi Zhuang, Qiandongnan Miao and Fujian She with other Chinese populations (Fig. 1C). Neighbor-Joining tree shows two separated clusters, one comprises western or northwestern Chinese ethnicities (Xinjiang Uyghur, Tibet Tibetan, two Mongolians and Qinghai Hui), the other consists of the remaining 17 populations. Our studied Bouyei, Han, and Miao are first respectively grouped with Guangxi Zhuang, Liangshan Yi and Guizhou Shui (Fig. 1D).

**Figure 2:**
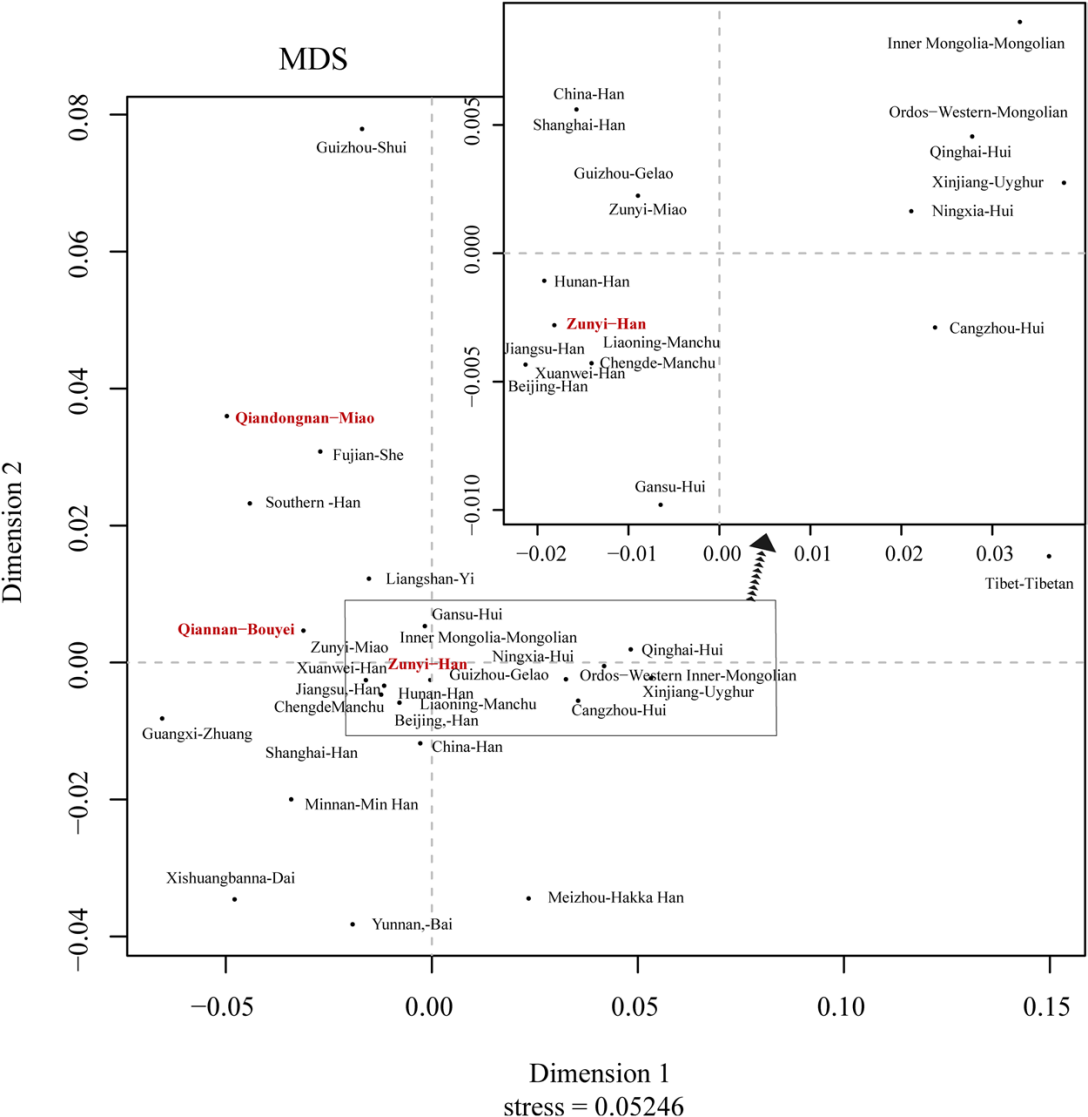
Multidimensional Scaling plots of our three investigated populations (bold and red) and 27 Chinese reference populations along ethnic and administrative boundaries based on PowerPlex Y23 haplotypes.

**Figure 3:**
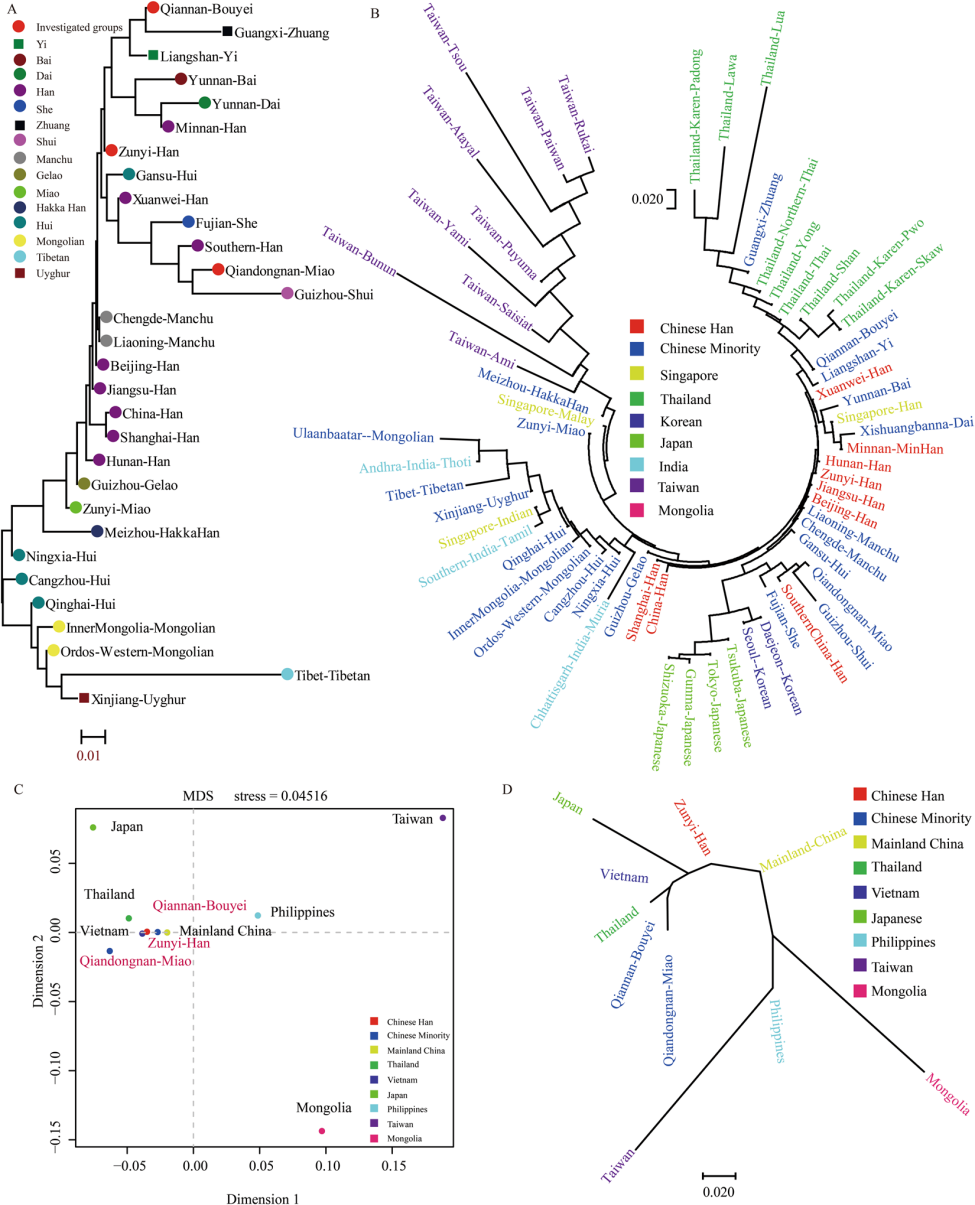
Genetic similarities and differences among our targets and reference populations along administrative or national boundaries. (**A**) The phylogenetic tree between the three studied populations and 22 Chinese populations based on Y-chromosomal haplotypes. (**B**) The Neighbor-Joining tree show the genetic affinity and divergence among 58 Asian populations; (**C**) Multidimensional Scaling plots of our studied populations and 7 Meta-populations based on Y-chromosomal haplotypes; (**D**) Phylogenetic relationship between seven Meta-populations and three investigated populations.

Subsequently, we calculate pairwise Rst values among Han Chinese populations and minorities. The Tibetan, as one high altitude adaptation residing in the Qinghai-Tibet Plateau, shows substantial genetic distinction from all other ethnicities with the corresponding pairwise genetic distances span from 0.0802 to 0.2344 (mean ± SD: 0.1616 ± 0.0416). The average Rst values reflecting individual population genetic differences range from 0.0302 at Zunyi Han to 0.1616 at Tibet Tibetan (Supplementary Table S9). According to the previous reported arbitrary threshold of larger than 0.05^23^, obvious substructures with other reference ethnicities are identified at Qinghai Hui, Ordos-Western Mongolian, Cangzhou Hui, Minnan Han, Yunnan Bai, Fujian She, Meizhou Hakka Han, Southern Han, Inner Mongolia Mongolian, Qiandongnan Miao, Guangxi Zhuang, Yunnan Dai, Xinjiang Uyghur, Guizhou Shui, and Tibet Tibetan. Figure 2 presents the cluster of genetically closely related populations and the dissimilar ones. Shui, Miao, She, Southern Han, Dai, Bai, Hakka Han, and Tibetan are dispersedly distributed in the MDS plot. Phylogenetic relationships among these 30 mainland Chinese populations^19,36–42^ are visualized using the Neighbor-Joining tree (Fig. 3A). The general population cluster is consistent with the aforementioned phylogenetic relationship reconstruction among the population along ethnic origin except for Han group. Han Chinese populations, with the exception of Xuanwei Han, Minnan Han, and Southern Han, are grouped together and subsequently grouped with Manchu and Gelao. Genetic differentiation based on the Y-chromosomal STR haplotypes among the mainland Chinese populations is observed between minority ethnicities, including most predominantly Tibetan, Shui, and Uyghur.

### Genetic differentiation along national or continental geographical divisions

To further explore the genetic background between the newly studied populations and 58 reference populations from all overall Asian^19,23,35–43^, we included 27 mainland Chinese populations, three Indian populations, four Japanese populations, two Korean populations, one Mongolian population, three Singaporean population, nine Taiwanese populations, and nine Thai populations in the comprehensive population comparisons. In pairwise population comparisons with our studied subjects, the Rst values span from −0.0003 (Xuanwei Han) to 0.4026 (Taiwan Tsou) in Zunyi Han, from 0.0079 (Northern Thai) to 0.4683 (Taiwan Tsou) in Qiandongnan Miao, from −0.0054 (Thailand Shan) to 0.4485 (Taiwan Tsou) in Qiannan Bouyei (Supplementary Table S10). Phylogenetic relationship among 61 Asian populations is constructed based on the Rst genetic matrixes using the Neighbor-Joining algorithm. In the dendrogram (Fig. 3B), Taiwanese populations combined with Meizhou Hakka Han form one cluster, and the remaining populations form the other cluster consisting of three sub-cluster. Thailand populations keep a genetic affinity with southwestern Chinese ethnic groups and constitute the first sub-cluster. Japanese and Korean populations have a close genetic relationship with North Chinese ethnicities and form the second sub-cluster. Northwestern Chinese populations are clustered with Indian and Mongolia Mongolian population and form the third sub-cluster. Our findings based on the Y-chromosomal STR haplotype data in East Asia demonstrated that genetic affinity is accompanied with close geographical positions (Taiwan and Meizhou, North China and Japan or Korea, Northwest China and Mongolia, as well as southwest China and Thailand), as well as closely similar ethnic origins (Tibeto-Burman populations and Hmong–Mien populations).

**Figure 4:**
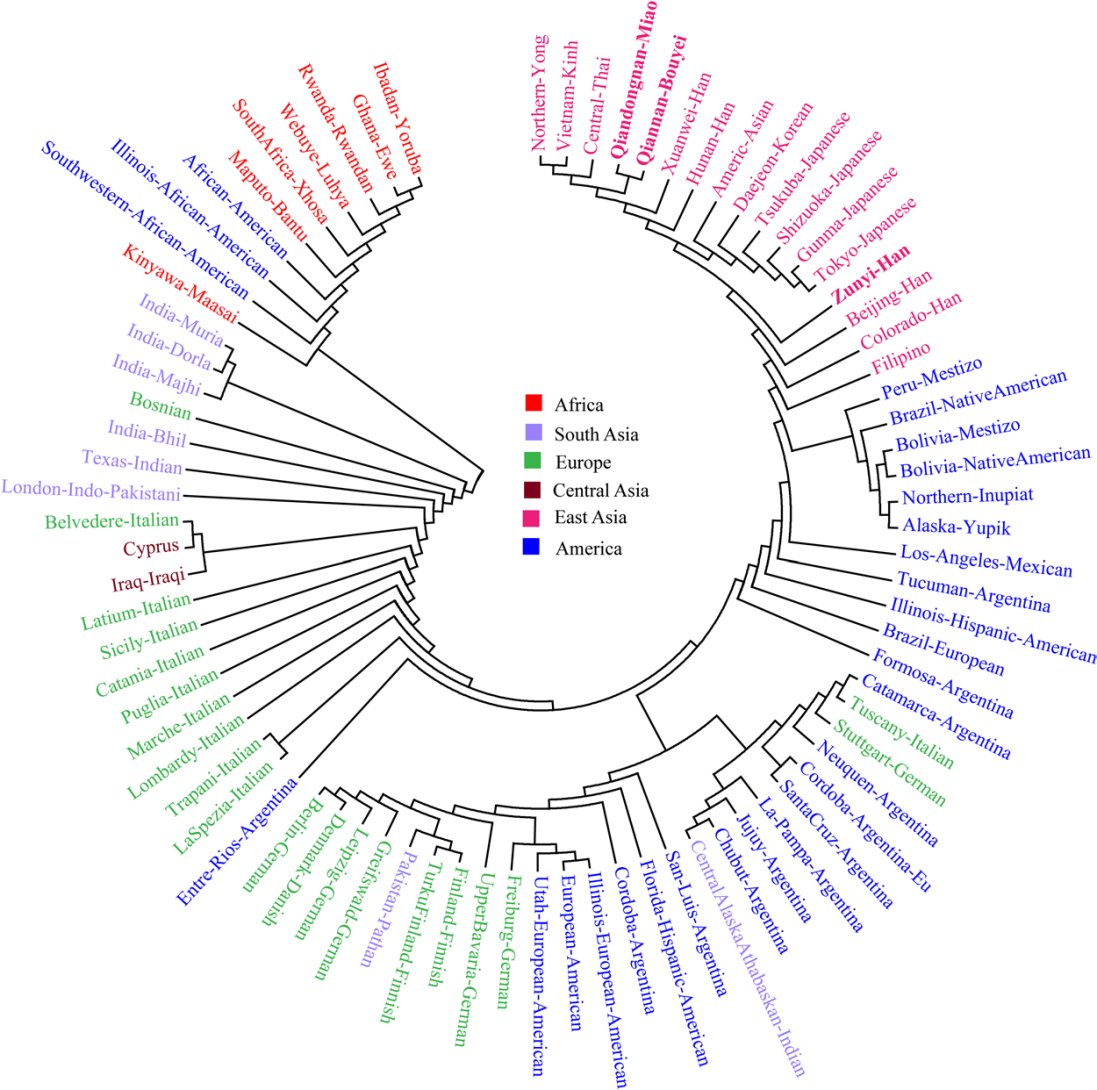
Phylogenetic tree constructed by the Neighbor-Joining method using the Mega 7.0 software based on Y-chromosomal STRs shows the phylogenetic relationship among three studied populations (red and bold) and 86 reference populations.

Calculation of Rst values between our studied subjects and seven meta-populations^19,23,35–43^ (combination on the basis of national or local boundaries) further reveal the partial population substructure between large-scale geographic divisions. As shown in Supplementary Table S11, the pairwise Rst values range from 0.0020 to 0.2257 for Zunyi Han, from 0.0171 to 0.3375 for Qiandongnan Miao, from 0.0016 to 0.2888 for Qiannan Bouyei. Taiwanese populations (292 haplotypes) show the substantial differences from all other reference populations with pairwise Rst values vary from 0.1117 to 0.3375 (mean ± SD: 0.2492 ± 0.0694). Multidimensional scaling results show a genetic cluster consisting of our subjects, Thailand, Vietnam, Mainland China, and Philippines. However, Japan is isolated and located in the upper left corner, Taiwan in the upper right corner and Mongolia in the lower right corner (Fig. 3C). Qiannan Bouyei and Qiandongnan Miao are first clustered with Thailand, and Zunyi Han is subsequently clustered with Japan and Mainland China in the phylogenetic relation reconstruction tree. Finally, haplotypes of 23 Y-STRs from 89 worldwide populations^23,25,40–48^ combined with our data are used to investigate the genetic divergence and similarities. The average of pairwise Rst values focused on Zunyi Han, Qiandongnan Miao and Qiannan Bouyei are 0.1445, 0.1922, and 0.1660, respectively. Five clear genetic affinity clusters can be identified: East Asian cluster, American cluster, European cluster, South Asian cluster, and African cluster (Fig. 4). Genetic substructure revealed by Y-chromosomal haplotype data is in accordance with our previous worldwide population structure investigation on the basis of ancestry informative single nucleotide polymorphisms^2,48^. Africa, as the origin of anatomically modern human harbors more genetic diversity than any other part of the world (especially with East Asians). Our results, as expected, demonstrated that our targeted studied populations keep the furthest genetic relationship with Africans.

## Discussions

### Forensic genetic characteristics and haplotype diversity

Y-STRs can be categorized into two main kinds on the basis of Y-STR mutation rates: Rapidly Mutating (RM) Y-STRs with mutation rates approximately 10^−2^ per locus per generation and slowly mutating (SM) Y-STRs with the low to midrange mutation rates (approximately∼10^−3^)^1,9,10,49^. SM Y-STRs have the advantages in the phylogenetic studies and providing investigative leads in the family searches^50^, however, RM Y-STRs are more suitable for forensic paternal lineage identification or other complex kinship identification^51^. PowerPlex^®^ Y23 system included all markers included in the previously developed systems (Minimal haplotype, PowerPlex^®^ Y and AmpFlSTR^®^ Yfiler) and other four SM Y-STRs (DYS481, DYS533, DYS549 and DYS643) and two RM Y-STRs (DYS570 and DYS576) included in the recently selected 13 rapidly mutation systems^20,51^. To accurately estimate the match probability of the Y-chromosomal haplotype from the crime scene to the perpetrator or infer the male biological ancestry of Chinese minority populations, more haplotype data and detailed genetic diversity form different populations are important. Besides, all forensic analysts, researchers focused on the historical and family genetics have commonly recognized that the appropriate grouping of haplotypes based on ethnically/geographically/linguistically different populations are necessary for the sensitivity of male-specific STRs to population differentiation^9,12,52,53^.

Thus, in the present study, we investigated the genetic polymorphisms/haplotype diversity and forensic characteristics of 23 Y-chromosomal STRs (21 single-copy Y-STR loci and one multi-copy locus. 23 Y-STRs) in 101 unrelated southwest Han Chinese individuals, 98 Bouyei individuals and 109 Miao individuals residing in Guizhou Province using the PowerPlex Y23 PCR amplification kit. DYS385a/b locus is the most diverse and polymorphic marker (GD > 0.9468) in three populations. The forensic diversity measures of RMP, DC, and HD in three newly genotyped Chinese populations, combined with the previously investigated Gelao^36^ and Yi^37^, demonstrated that 23 Y-STRs are highly informative and polymorphic and could be served as a useful and interesting tool in forensic practical applications. Additionally, this study enriches the Y-STR database of the southwest Chinese ethnic populations and provides the first batch of Y-chromosome STR profiling data of these three underrepresented Chinese populations to the Y Chromosome Haplotype Reference Database (YHRD), which is essential to assist the calculation and interpretation of match probabilities in forensic male lineage identification, as well as characterizing population male genetic history^12,52,53^.

### Population genetic characteristics and phylogenetic relationships

Nothnagel *et al*. recently revisited the Chinese male genetic landscape on the basis of 38,000 17-Y-STR haplotypes and found Han Chinese populations are homogeneity and genetic differentiation exists among Minorities (Tibetans and Kazakhs) and Han Chinese^23^. However, there are a large number of population migration and genetic admixture (Mongol empire in Eurasian, Arab slave trade and Bantu expansion in Africa, first millennium CE migrations and prehistory colonialism in Europe) after anatomically modern human migrated out of Africa. Besides, Abundant evolutionary forces (genetic drift, introgression and natural selection) also has shaped the genetic landscapes nowadays^8,23,54,55^. Y-chromosomal STRs are important and indispensable to explore the origin of modern humans, tracing the migration trajectories and timing of ancient human, and inferring the male genetic genealogy evolution, as well as dissecting the population stratification for constructing regional-effective forensic reference database and reasonably designing case-control studies in the whole genome association studies to avoid false positive results. Thus, we conducted a more comprehensive population genetic comparisons based on more Y-STRs (23) to dissect the genetic relationships of worldwide populations and Chinese nationwide populations. In this study, we conducted the population comparisons to investigate the detailed genetic background of our focuses (Zunyi Hans, Qiandongnan Miaos and Qiannan Bouyeis) as well as explore the genetic relationships among 30 mainland Chinese populations (Han Chinese and minority populations)^19,23,35–43^, 58 Asian populations^19,23,35–43^ and 89 worldwide populations^19,23,35–44^. Our results demonstrated that Qiannan Bouyei has a close genetic affinity with Guangxi Zhuang, as well as Zunyi Han with Hunan Han, and Qiandongnan Miao with Guizhou Shui. Additionally, significant genetic distinctions have existed among Hans, Uyghurs, Tibetan and Taiwanese populations. Population structure analysis revealed a strong association between genetic distance and geographical or ethnic affinity.

Gao *et al*. have investigated the genetic diversity of the 23 Y-STRs and population structure among 12 worldwide populations with sample sizes varying from 9 to 92^38^ and Purps *et al*. analyzed the Y-chromosomal haplotype diversity of 19,630 individuals from 129 different populations in 51 countries^19^. In this study, we conducted genetic studies on the basis of 308 individuals from 3 populations. Considering the relatively small sample size, more attention should be paid in the forensic practices and population genetic applications. Inter- and intra-populations structure between the three focuses (Han, Miao and Bouyei) and other reference populations along different geographical/ethnically divisions revealed that the Zunyi Han is genetically close to other geographically adjacent Han Chinese populations and keeps the remote genetic relationships with Miao and Bouyei populations. Bouyei population has a stronger genetic homogeneity with Zhuang population, both of them belong to the Tai-Kadai-speaking populations. We identified the genetic stratification among Han populations within the Sino-Tibetan-speaking populations, and Bouyeis and Zhuang populations within the Tai-Kadai-speaking populations. However, genetic affinities among different language-family-speaking populations are also observed, such as Hmong-Miens-speaking population of Miao and Tai-Kadai-speaking population of Shui. Significant genetic differences along continental boundaries based on the Y-chromosome generations is also identified. Worldwide population relationship patterns are consistent with the geographical categories. The present results emphasize the common paternal ancestry of same language family populations, and geographical isolation and paternal residence play a pivotal role in population structure reconstruction. To better disentangle the genetic structure and population history of Han, Miao and Bouyei in the natural processes (mutation, genetic drift, migration and selection) and elucidating genetic perspectives with other linguistically and geographically related populations, further studies based on the maternal mitochondrial DNA and whole genome sequence data or high-density chips data should be considered.

## Conclusions

This study investigated the Y-chromosomal STR haplotype distributions in three Guizhou ethnicities in the southwestern China and submitted the first batch of Y-STR haplotype data of Zunyi Han (YA004233), Qiandongnan Miao (YA004235) and Qiannan Bouyei (YA004236) for forensic, genealogical, and ethnic evolutionary researches. The HD in the aforementioned three populations are 0.99990, 0.99983, and 0.99979, respectively, and DC values are 0.9902, 0.9908, and 0.97959, respectively. Comprehensive population comparisons along with ethnic divisions, administrative divisions, and national/continental boundaries were performed using AMOVA, MDS, and Neighbor-Joining phylogenetic relationship reconstructions. Overall, Qiannan Bouyei has a genetic relationship with Guangxi Zhuang. And Zunyi Han and Qiandongnan Miao respectively have the genetic affinity with Hunan Han and Guizhou Shui. Genetic structures of our studied three populations are significantly different from Chinese minority ethnicities (especially in Tibetan and Uyghur) and Taiwanese populations in the East Asian. Worldwide population structure demonstrated that five population sub-structures can be dissected based on the Y-STR haplotype data in accordance with continental divisions.

## Materials and Methods

### Ethics Statement

All of the experimental procedures in this study were strictly followed the humane and ethical research principles. All participants signed the written informed consent before sample collection. Our study design was approved by the Medical Ethics Committee of Zunyi medical university and Sichuan University.

### Sample collection and DNA preparation

Peripheral blood samples were collected from 308 unrelated healthy Chinese individuals from three Chinese major population groups in Guizhou Province, southwest China, including: 98 Bouyei individuals residing in Qiannan District, 101 Han individuals recruited from Zunyi District, 109 Miao individuals residing in Qiandongnan District. One milliliter of blood was obtained in tubes with EDTA. The ancestors of all subjects must live in the present region at least three generations. The QIAamp DNA Blood Mini Kit (Qiagen, Hilden, Germany) was used to extract the genomic DNA based on the manufacturer’s recommendations. A 7500 Real-time PCR system (Thermo Fisher Scientific) was employed to determine the DNA concentration using Quantifiler Human DNA Quantification Kit. The DNA was diluted to 1 ng/μL and stored at −20 °C until amplification.

### Multiplex amplification and genotyping

Twenty-three Y-STR loci (DYS533, DYS438, DYS437, DYS570, DYS635, DYS390, DYS439, DYS392, DYS643, DYS576, DYS389I, DYS448, DYS389II, DYS19, DYS391, DYS481, DYS549, DYS393, DYS458, DYS385a/b, DYS456, and GATA-H4) included in the PowerPlex Y23 System were co-amplified in one multiplex PCR reaction on a ProFlex PCR System (Thermo Fisher Scientific) according to the manufacturer’s instructions. In brief, 25 μL PCR reaction volume, consisting of 0.5 μL of template DNA, 5 μL of master mix and 2.5 μL of primer pair mix was employed. PCR cycling conditions were 96 °C for 1 min, followed by 28 cycles of 94 °C for 10 sec, 61 °C for 1 min, 72 °C for 30 sec, and a final extension at 60 °C for 20 min. Separation and detection of PCR amplified products were conducted using capillary electrophoresis on a 3500 Genetic Analyzers (Applied Biosystems, Foster City, CA, USA) with 36 cm capillary array and POP-4 polymer. 1 μL amplified product was added to deionized Hi-Di formamide (10 μL) with 1 μL CC5 ILS 500 Y23 size standard (Thermo Fisher Scientific). Capillary electrophoresis was conducted with the injection voltage of 1.2 kV and injection time of 15 sec. Allele designation was conducted using the software of GeneMapper ID-X v.1.4 by comparison with the allele ladder provided by the corresponding kit, which was followed by the DNA Commission of the International Society of Forensic Genetics (ISFG)^56^.

### Quality control

We carried out all of our experimental procedures in forensic genetic laboratory in the Department of Forensic Biology, West China School of Basic Science and Forensic Medicine, Sichuan University, which is accredited with the China National Accreditation Service for Conformity Assessment (CNAS) and ISO 17025. The recommendations published by the DNA Commission of the International Society for Forensic Genetics (ISFG) were followed in the overall experimental procedure^57^. The positive of control DNA 2800 M and negative control of ddH_2_O in each batch of genotyping were conducted. The genotype data of three Chinese ethnic groups were submitted to the Y chromosome haplotype reference database (YHRD)^12,52,53^ (http://www.yhrd.org) and received the following accession number YA004233 (Zunyi Han), YA004235 (Qiandongnan Miao) and YA004236 (Qiannan Bouyei).

### Statistical analysis

Allele frequencies of 23 Y-STR loci and haplotype frequencies were calculated using the direct counting method. Forensic statistical parameters of gene diversity (GD) and haplotype diversity (HD) were calculated using the Nei’s formula^58,59^:

1 $${\rm{GD}}=\frac{{N}_{a}}{{N}_{a-1}}(1-{\sum }^{}{P}_{ai}^{2}),$$

or

2 $${\rm{HD}}=\frac{{N}_{h}}{{N}_{h-1}}(1-{\sum }^{}{P}_{hi}^{2}),$$

in which *N*_*a*_ and *N*_*h*_ respectively denote the total number of the tested samples and haplotypes, and *P*_*ai*_ and *P*_*hi*_ respectively mean the allele frequency of the *i*_*th*_ allele of corresponding locus and *i*_*th*_ haplotype. The discrimination capacity (DC) was assessed as using the following formula:

3 $${\rm{DC}}=\frac{A}{{{\rm{N}}}_{h}},$$

where *A* and *N*_*h*_ respectively means the number of distinct haplotypes in one population and total observed haplotypes. The random match probability (RMP) was determined as:

4 $${\rm{RMP}}={\sum }^{}{P}_{hi}^{2},$$

in which *P*_*hi*_ denotes the *ith* haplotype frequency. Comprehensive populations comparisons at different scales based on Y-chromosomal STR haplotype data are performed to investigate genetic similarities and differences between our studied populations and eight Han Chinese populations, nineteen Chinese minority ethnicities, fifty-eight Asian populations, seven Meta-populations, and eighty-nine worldwide populations^23,25,40–48^, respectively. Pairwise Rst genetic distances are calculated via the online tool in the YHRD using the analysis of molecular variance (AMOVA), and Multidimensional Scaling plots (MDS) based on different Rst genetic matrixes are also performed using the online tool in the YHRD^12,52,53^. According to the constraints in the process of AMOVA and MDS calculation, all haplotypes with unspecified alleles, intermediate alleles, null-alleles, triplicated or duplicated alleles were removed, and DYS389I were substracted from DYS389II for the Multi copy locus. Finally, phylogenetic relationships are reconstructed on the basis of Rst genetic matrix using the Molecular Evolutionary Genetics Analysis 7.0 (MEGA 7.0) software^60^.

## Electronic supplementary material

Supplementary Tables S1–S11.
