# Supplementary material for: Genetic diversities and phylogenetic analyses of three Chinese main ethnic groups in southwest China: A Y-Chromosomal STR study
Source: Sci Rep. 2018 Oct 18;8:15339. doi: 10.1038/s41598-018-33751-x (PMC6193932; doi:10.1038/s41598-018-33751-x)
Supplement: Supplementary file 1 — Supplementary Tables S1–S11. [file 41598_2018_33751_MOESM1_ESM.pdf]

Supplementary Tables S1-S11

Genetic diversities and phylogenetic analyses of three Chinese main ethnic groups in southwest China: a Y-Chromosomal STR study

Pengyu Chen[1,2+], Guanglin He[3+], Xing Zou[4], Xin Zhang[5], Jida Li[6], Zhisong Wang[6], Hongyan Gao[1,2], Li Luo[1,2], Zhongqing Zhang[1,2], Jian Yu[1,2], Yanyan Han[6]*

[1]Center of Forensic Expertise, Affiliated Hospital of Zunyi Medical University, Zunyi, Guizhou, China

[2]Department of Forensic genetics, School of Forensic Medicine, Zunyi Medical University, Zunyi, Guizhou, China

[3]Institute of Forensic Medicine, West China School of Basic Medical Sciences & Forensic Medicine, Sichuan University, Chengdu, Sichuan, China

[4]Department of Forensic Medicine, College of Basic Medicine, Chongqing Medical University, Chongqing, China

[5]People's Hospital of Wuxi County, Chongqing, China

[6]School of Public Health, Zunyi Medical University, Zunyi, Guizhou, China

+ These authors contributed equally to this work.

* Correspondence and requests for materials should be addressed to Yanyan Han (hanyanyan1984@126.com)

Supplementary Table S1. 23 Y-STR haplotypes of Chinese Bouyei minority group from Qiannan prefecture in Guizhou Province (98).

ID	DYS19	DYS389I	DYS389II	DYS390	DYS391	DYS392	DYS393	DYS385	DYS438	DYS439	DYS437	DYS448	DYS456	DYS458	DYS635	YGATAH4	DYS576	DYS481	DYS549	DYS533	DYS570	DYS643	Haplotype	n
M001	13	13	29	24	9	14	14	14,21	12	11	14	19	16	18	22	10	16	24	12	12	21	10	HD1	1
M002	13	14	30	25	9	15	13	15,22	12	12	14	20	15	17	22	10	20	23	12	12	22	10	HD2	1
M003	14	12	28	22	10	14	12	13,19	11	11	14	20	16	17	20	12	18	23	13	11	19	11	HD3	1
M004	14	12	28	24	10	14	12	13,17	11	12	15	20	15	19	20	12	20	24	12	11	16	12	HD4	1
M005	14	12	28	24	10	14	12	13,17	11	12	15	20	15	20	20	12	20	24	12	11	16	12	HD5	1
M006	14	12	28	24	10	14	12	14,17	11	12	15	20	15	20	20	12	20	24	12	11	16	12	HD6	1
M007	14	12	28	24	10	14	12	12,17	11	12	15	20	16	18	20	11	20	24	12	11	16	12	HD7	1
M008	14	12	28	24	10	14	12	12,17	11	12	15	20	16	20	20	12	21	24	12	11	16	12	HD8	1
M009	14	12	28	24	11	14	12	13,19	11	12	15	19	15	20	22	12	16	24	12	11	17	11	HD9	1
M010	14	12	28	25	10	14	12	13,17	11	13	15	20	15	18	20	12	19	23	14	11	17	12	HD10	1
M011	14	12	29	23	10	12	12	12,16	10	13	15	19	16	19	21	12	20	22	12	11	16	11	HD11	1
M012	14	12	29	24	10	11	13	15,15	11	10	15	20	14	18	19	12	18	25	12	12	17	10	HD12	1
M013	14	12	29	24	10	11	13	15,15	11	12	15	19	14	16	19	13	19	25	13	12	17	10	HD13	1

M014	14	12	30	24	10	11	13	15,15	11	12	15	19	14	15	19	13	19	25	14	12	17	10	HD14	1
M015	14	12	30	24	10	11	13	15,15	11	12	15	19	14	16	19	13	18	25	13	12	17	10	HD15	1
M016	14	12	30	24	10	11	13	15,15	11	12	15	19	14	16	19	13	19	25	13	12	17	10	HD16	1
M017	14	12	30	24	10	11	13	15,15	11	12	15	19	14	16	19	13	19	25	14	12	17	10	HD17	1
M018	14	12	30	24	10	11	13	15,15	11	12	15	19	14	16	19	13	20	25	12	12	17	10	HD18	1
M019	14	12	30	24	10	11	13	14,15	11	12	15	19	14	16	19	13	20	25	15	12	17	10	HD19	1
M020	14	12	30	24	10	11	13	15,15	11	12	15	19	14	16	19	14	20	26	14	12	17	10	HD20	1
M021	14	12	30	24	10	11	13	15,15	11	12	15	19	14	16	20	13	18	25	13	12	17	10	HD21	1
M022	14	12	30	24	10	11	13	15,15	11	12	15	19	14	17	19	12	19	25	13	12	17	10	HD22	1
M023	14	12	30	24	10	11	13	15,15	11	12	15	19	14	17	19	13	19	25	14	12	17	10	HD23	1
M024	14	12	30	24	10	14	12	13,17	11	11	15	20	15	17	20	12	18	24	12	11	17	12	HD24	1
M025	14	12	31	24	10	11	12	15,16	11	11	15	19	14	16	19	12	19	25	12	12	18	10	HD25	1
M026	14	13	28	23	10	14	13	13,14	10	12	14	18	15	14	19	11	17	25	13	11	20	11	HD26	1
M027	14	13	28	23	10	14	13	13,14	10	12	14	18	15	15	19	12	17	24	13	11	20	11	HD27	1
M028	14	13	28	23	10	14	13	13,14	10	12	14	18	15	15	19	12	19	24	13	11	20	12	HD28	1

M029	14	13	29	23	10	14	13	13,14	10	12	14	18	15	15	19	12	17	24	13	11	20	11	HD29	1
M030	14	13	29	24	10	14	12	13,18	11	12	15	20	15	20	20	12	17	24	12	11	16	11	HD30	1
M031	14	13	30	24	10	11	13	15,15	11	12	15	19	14	17	19	13	19	25	14	12	17	10	HD30	1
M032	14	13	30	24	10	11	13	15,15	11	13	15	19	14	17	19	13	19	25	14	12	17	10	HD31	1
M033	14	13	32	24	10	11	13	17,18	10	11	14	20	15	16	20	12	19	24	12	11	16	10	HD32	1
M034	14	14	30	22	10	14	13	13,14	10	12	14	18	16	15	20	12	17	25	12	12	20	11	HD33	1
M035	14	14	31	23	10	14	14	11,12	11	11	14	19	16	15	24	11	17	25	12	13	19	10	HD34	1
M036	15	10	26	21	10	11	12	11,17	10	11	14	20	15	17	25	11	18	28	12	11	19	9	HD35	1
M037	15	12	28	21	10	11	12	11,19	10	11	14	20	15	18	25	11	18	27	12	11	19	10	HD36	1
M038	15	12	28	21	11	11	12	11,19	10	11	14	20	15	18	25	11	18	27	12	11	19	10	HD36	1
M039	15	12	28	23	10	14	14	14,14	10	11	14	16	19	18	21	11	17	27	13	11	18	10	HD37	1
M040	15	12	28	23	11	14	13	13,13	10	12	14	18	16	13	21	13	17	24	13	11	20	11	HD38	1
M041	15	12	28	24	10	13	12	12,19	11	11	14	19	14	18	21	12	18	25	12	11	18	10	HD39	1
M042	15	12	28	24	10	13	15	14,16	10	11	14	18	15	18	23	10	18	23	11	10	16	10	HD40	2
M043	15	12	28	24	10	13	15	14,16	10	11	14	18	15	18	23	10	18	23	11	10	16	10	HD41	

M044	15	12	28	24	10	14	12	13,16	11	12	15	20	15	20	20	12	19	24	12	11	15	12	HD42	1
M045	15	12	28	25	10	13	12	12,20	10	11	14	19	14	17	20	13	19	26	13	11	19	10	HD43	1
M046	15	12	28	25	10	13	12	12,21	10	12	15	20	14	16	21	12	17	27	12	12	20	11	HD43	1
M047	15	12	28	25	9	13	12	12,21	10	9	14	19	14	17	21	12	21	26	13	11	17	10	HD43	1
M048	15	12	28	26	10	13	12	12,18	10	12	14	19	15	21	23	11	18	23	13	11	17	10	HD44	1
M049	15	12	29	24	9	14	12	13,17	9	12	15	18	15	18	21	11	20	24	13	12	16	12	HD45	1
M050	15	12	30	23	10	14	14	13,14	10	12	14	18	16	15	21	12	17	25	12	12	20	11	HD46	1
M051	15	12	31	22	10	14	13	12,13	10	13	14	18	15	13	21	12	16	25	12	12	19	11	HD47	1
M052	15	13	28	24	11	14	14	15,21	9	12	14	18	15	15	21	11	17	24	12	10	16	12	HD48	1
M053	15	13	28	25	10	13	13	16,18	10	12	14	18	15	16	22	11	17	21	13	11	18	13	HD49	1
M054	15	13	29	24	11	10	12	16,16	10	11	14	19	15	22	20	11	17	28	11	12	20	9	HD50	1
M055	15	13	29	25	10	13	13	16,18	10	12	14	18	15	16	22	11	18	21	13	11	18	13	HD51	1
M056	15	13	29	25	10	13	13	16,17	10	12	14	18	15	16	23	11	17	21	13	11	18	13	HD52	1
M057	15	13	29	25	10	13	13	16,18	10	13	14	18	15	16	22	11	18	21	13	11	18	13	HD53	1
M058	15	13	29	25	10	13	13	16,18	11	12	14	18	15	16	22	11	20	21	13	11	18	13	HD54	1

M059	15	13	29	25	11	13	13	16,18	11	12	14	18	15	15	23	11	18	21	13	11	17	13	HD55	1
M060	15	13	30	23	10	11	16	11,18	10	12	14	22	15	16	22	11	17	24	12	12	16	9	HD56	1
M061	15	13	30	24	10	13	14	13,14	10	11	14	18	16	17	21	11	19	23	12	10	17	13	HD57	1
M062	15	13	30	24	11	13	14	13,20	10	13	14	18	15	18	24	11	18	23	11	10	13	12	HD58	1
M063	15	13	30	25	10	13	14	12,17	10	11	14	18	15	15	21	12	18	24	12	11	19	11	HD59	1
M064	15	13	30	25	11	13	13	16,19	11	12	14	18	15	15	23	11	19	21	13	11	18	13	HD60	1
M065	15	13	31	23	10	11	13	16,17	10	13	15	19	14	19	19	12	18	26	13	11	18	10	HD61	1
M066	15	14	29	23	10	11	14	11,19	10	10	14	21	15	18	22	11	17	26	12	12	15	9	HD62	1
M067	15	14	30	23	11	14	13	11,12	11	11	14	20	16	15	21	12	19	22	12	12	19	9	HD63	1
M068	15	14	30	24	11	13	14	13,13	10	12	14	18	14	18	22	10	19	22	12	10	20	10	HD64	1
M069	15	14	31	23	10	13	12	12,19	10	11	14	19	14	17	22	11	19	23	12	11	17	11	HD65	1
M070	15	14	31	23	10	13	14	15,16	10	11	14	18	15	14	24	12	18	23	12	9	16	10	HD66	1
M071	15	14	31	24	10	13	14	14,18	11	11	14	18	15	17	20	10	19	21	12	10	16	12	HD66	1
M072	16	11	27	24	10	13	12	13,22	10	12	15	20	15	19	22	12	20	22	11	11	19	10	HD67	1
M073	16	12	27	24	10	13	12	13,22	10	11	15	20	16	19	23	12	17	21	12	11	20	10	HD68	1

M074	16	12	27	24	10	13	12	13,22	10	11	15	20	16	19	23	12	18	21	11	11	20	10	HD69	1
M075	16	12	27	24	10	13	12	13,22	10	11	15	20	16	20	23	12	18	21	11	12	19	10	HD70	1
M076	16	12	27	24	10	13	12	13,22	10	11	15	20	16	20	23	12	18	21	11	12	20	10	HD71	1
M077	16	12	27	24	10	13	12	13,22	10	11	15	20	16	20	23	12	18	21	12	11	20	10	HD72	1
M078	16	12	27	24	10	13	12	13,22	10	11	15	20	16	21	23	12	18	21	11	12	20	10	HD73	1
M079	16	12	27	24	10	14	12	13,23	10	11	15	20	16	19	23	12	18	21	12	11	20	10	HD74	1
M080	16	12	28	24	10	13	12	13,22	10	10	15	20	16	19	23	12	18	21	12	11	20	10	HD75	1
M081	16	12	28	24	10	13	12	13,22	10	11	15	20	15	19	23	12	18	21	11	12	20	9	HD76	1
M082	16	12	28	25	10	13	12	12,20	10	12	14	19	14	17	20	13	19	26	13	11	18	10	HD77	1
M083	16	12	30	23	10	14	13	12,14	11	11	14	20	15	16	21	12	16	25	12	12	19	11	HD78	1
M084	16	13	29	24	11	13	14	14,18	10	13	14	18	15	17	22	11	19	23	12	10	16	11	HD78	1
M085	16	13	29	24	11	13	14	14,18	10	13	14	18	15	18	21.3	11	19	23	12	10	16	11	HD79	1
M086	16	13	29	24	11	13	14	14,18	10	13	14	18	15	18	22	11	19	23	12	10	16	11	HD80	2
M087	16	13	29	24	11	13	14	14,18	10	13	14	18	15	18	22	11	19	23	12	10	16	11	HD81	
M088	16	13	30	24	10	13	14	13,14	10	11	14	18	17	17	21	11	19	23	12	10	17	13	HD82	1

M089	16	13	30	24	11	13	14	13,18	10	12	14	18	15	18	23	11	18	23	12	10	17	12	HD83	1
M090	16	13	30	25	10	13	14	14,18	10	11	14	18	15	17	22	10	18	23	11	10	17	13	HD84	1
M091	16	14	30	23	11	13	14	12,21	10	11	14	18	15	19	24	10	18	23	12	10	16	12	HD85	1
M092	17	12	28	24	10	13	12	13,21	10	10	15	20	15	18	23	12	17	22	12	11	20	10	HD86	1
M093	17	12	28	24	10	13	12	13,22	10	11	15	20	15	19	23	12	18	22	12	11	20	11	HD87	1
M094	17	12	28	24	10	13	12	13,22	10	11	15	20	15	19	23	12	19	22	12	11	20	11	HD88	1
M095	17	12	28	24	10	13	12	13,22	10	11	15	20	17	19	23	12	18	22	12	11	19	10	HD89	1
M096	17	12	28	24	10	13	13	13,24	10	11	15	20	15	19	23	12	17	22	12	11	20	10	HD90	1
M097	17	13	30	23	11	12	12	12,16	10	12	15	19	16	18	20	12	19	22	12	11	17	11	HD91	1
M098	17	13	30	25	10	13	12	13,19	10	12	14	20	15	19	22	11	17	24	12	11	18	11	HD92	1

Supplementary Table S2. The raw haplotype data of 23 Y-STRs included in PowerPlex Y23 kit in 101 Zunyi Han individuals.

ID	DYS19	DYS389I	DYS389II	DYS390	DYS391	DYS392	DYS393	DYS385	DYS438	DYS439	DYS437	DYS448	DYS456	DYS458	DYS635	YGATAH4	DYS576	DYS481	DYS549	DYS533	DYS570	DYS643	Haplotype	n
M001	13	12	28	23	10	12	12	12,17	10	13	15	20	15	19	19	11	20	22	12	11	17	11	HD1	1
M002	13	12	28	23	11	12	12	12,16	10	13	15	18	15	18	19	12	18	23	12	11	18	11	HD2	1
M003	13	13	29	24	9	14	14	15,20	12	11	14	19	18	18	22	10	18	25	12	12	20	10	HD3	1
M004	13	13	29	25	10	13	15	14,20	10	12	14	18	15	16	21	11	18	23	14	11	17	12	HD4	1
M005	13	14	30	24	9	15	14	15,20	12	11	14	19	16	17	22	10	20	24	12	12	21	10	HD5	1
M006	14	12	27	23	10	14	12	13,15	11	11	15	20	15	16	20	11	19	23	12	11	17	12	HD6	1
M007	14	12	27	23	10	16	12	13,19	11	11	14	20	16	17	20	12	19	24	12	11	18	11	HD7	1
M008	14	12	27	24	10	13	12	15,18	11	13	15	20	15	18	20	12	17	23	12	10	19	12	HD8	1
M009	14	12	27	24	10	14	12	13,17	11	13	15	20	15	19	21	12	18	23	12	11	19	11	HD9	1
M010	14	12	28	24	10	14	12	13,19	11	12	14	20	16	18	21	13	17	20	12	11	16	11	HD10	1
M011	14	12	28	24	10	14	12	15,19	11	12	15	20	16	18	20	11	19	23	12	11	17	11	HD11	1
M012	14	12	28	25	10	13	12	13,20	10	11	15	20	15	18	25	12	19	25	11	12	19	11	HD12	1
M013	14	12	28	25	10	13	12	13,19	10	12	15	20	15	16	23	12	19	27	13	11	19	11	HD13	1

M014	14	13	28	24	11	14	13	11,12	10	12	14	18	15	17	21	12	16	24	13	12	19	11	HD14	1
M015	14	13	29	23	10	14	12	13,19	11	12	15	20	15	17	20	11	21	23	13	12	22	11	HD15	1
M016	14	13	29	23	10	14	13	13,13	10	12	14	18	15	16	19	12	16	24	12	11	19	11	HD16	1
M017	14	13	29	23	11	14	13	11,12	11	12	14	19	14	15	24	12	19	22	13	12	16	10	HD17	1
M018	14	13	29	24	10	11	12	14,14	10	10	14	17	15	17	21	11	18	27	12	12	20	10	HD18	1
M019	14	13	29	24	10	11	13	14,14	10	12	14	19	16	18	24	11	19	17	12	11	16	9	HD19	1
M020	14	13	29	24	10	14	11	14,17	11	11	15	20	15	15	21	12	16	25	12	11	19	10	HD20	1
M021	14	13	30	23	10	14	12	13,19	11	11	14	20	15	17	21	11	18	23	11	11	19	11	HD21	1
M022	14	13	30	23	10	14	12	13,19	11	12	14	20	14	19	20	12	20	23	12	12	18	12	HD22	1
M023	14	14	30	22	10	14	13	11,12	10	10	14	19	16	19	22	12	17	21	12	12	19	11	HD23	1
M024	14	14	30	23	11	15	13	11,14	11	10	14	19	14	18	21	12	16	20	12	11	20	11	HD24	1
M025	14	14	30	24	10	14	12	14,19	11	12	14	20	15	15	20	12	18	25	12	11	18	11	HD25	1
M026	15	12	27	23	11	14	13	13,14	10	11	14	18	17	16	19	12	17	25	12	11	19	11	HD26	1
M027	15	12	27	25	10	13	12	14,18	10	12	14	20	14	18	22	11	16	23	14	11	18	10	HD27	1
M028	15	12	27	25	10	13	13	12,12	11	13	14	20	15	17	21	11	16	24	14	11	17	12	HD28	1

M029	15	12	28	22	10	15	13	13,13	10	12	14	19	15	15	20	12	16	24	13	12	19	11	HD29	1
M030	15	12	28	23	10	12	12	12,17	10	12	15	19	15	17	19	12	20	22	14	11	18	12	HD30	1
M031	15	12	28	23	10	12	12	12,17	10	12	15	19	15	18	20	11	18	22	11	11	18	11	HD31	1
M032	15	12	28	23	10	14	14	13,13	10	11	14	18	15	16	20	12	16	24	12	11	18	11	HD32	1
M033	15	12	28	23	11	12	12	12,16	10	13	15	19	16	18	20	12	17	21	12	11	19	11	HD33	1
M034	15	12	28	24	10	13	12	12,20	10	11	14	19	14	18	21	12	18	26	12	11	18	10	HD34	1
M035	15	12	28	24	10	13	12	14,14	10	12	14	19	13	18	22	11	18	23	13	12	17	9	HD35	1
M036	15	12	28	24	10	13	12	12,16	10	12	15	19	15	15	17	12	18	23	14	11	16	11	HD36	1
M037	15	12	28	24	10	14	12	13,16	11	13	14	20	14	17	22	12	20	23	11	11	16	11	HD37	1
M038	15	12	28	24	10	14	13	13,14	10	11	14	18	17	15	19	12	18	26	12	11	19	11	HD38	1
M039	15	12	28	25	10	10	12	16,17	10	11	14	19	15	20	20	12	17	28	11	13	21	9	HD39	1
M040	15	12	28	25	10	13	13	13,14	10	11	15	20	14	17	23	11	18	28	11	12	18	11	HD40	1
M041	15	12	28	25	9	13	7	14,18	10	11	14	20	14	17	23	12	16	24	14	11	19	10	HD41	1
M042	15	12	29	23	10	12	12	13,17	10	11	14	19	15	17	19	12	18	21	12	11	16	11	HD42	1
M043	15	12	29	23	10	12	12	12,16	10	11	14	19	15	18	19	12	21	21	12	11	16	11	HD43	1

M044	15	12	29	23	10	14	12	12,17	9	12	15	18	15	18	21	11	20	24	12	12	16	12	HD44	1
M045	15	12	29	23	10	16	13	13,14	10	13	14	17	16	16	21	11	20	25	12	11	20	11	HD45	1
M046	15	12	29	23	11	14	13	13,13	10	11	14	18	15	15	19	12	18	25	11	11	19	11	HD46	1
M047	15	12	29	23	11	14	13	13,14	10	11	14	18	18	15	19	11	19	25	13	11	19	11	HD47	1
M048	15	12	29	23	11	14	13	13,14	10	14	14	18	17	15	21	12	18	25	12	12	20	11	HD48	1
M049	15	12	29	24	10	11	14	14,18	11	12	14	18	15	18	20	10	20	23	11	10	14	12	HD49	1
M050	15	12	29	25	10	13	12	13,19	10	12	14	19	13	19	23	12	18	25	14	11	18	11	HD50	1
M051	15	12	30	23	10	14	12	13,14	11	12	14	18	15	15	21	12	16	26	12	12	20	11	HD51	1
M052	15	12	30	23	11	12	12	12,12	10	12	15	18	15	18	19	12	20	22	12	12	16	11	HD52	1
M053	15	12	30	23	11	12	12	12,16	10	12	15	19	16	20	22	12	18	22	12	10	17	11	HD53	1
M054	15	12	30	24	9	14	12	12,17	9	13	15	18	15	17	21	11	18	24	13	12	16	12	HD54	1
M055	15	13	28	24	10	13	12	13,21	10	12	15	19	15	19	22	11	18	27	12	11	19	11	HD55	1
M056	15	13	28	25	10	13	14	12,12	10	11	14	18	15	18	21	11	19	23	12	10	18	12	HD56	1
M057	15	13	28	25	10	13	14	12,21	10	12	14	18	15	16	22	12	17	21	13	10	20	11	HD57	1
M058	15	13	29	23	11	13	13	12,22	10	11	14	18	15	18	25	10	17	23	12	11	16	12	HD58	1

M059	15	13	29	23	11	13	14	12,20	10	11	14	18	15	18	24	10	17	23	12	10	16	12	HD59	1
M060	15	13	29	23	12	13	14	12,20	10	11	15	18	15	18	24	10	17	23	11	10	16	12	HD60	1
M061	15	13	29	24	10	13	12	14,18	10	12	14	18	15	18	22	10	18	23	12	11	17	12	HD61	2
M062	15	13	29	24	10	13	12	14,18	10	12	14	18	15	18	22	10	18	23	12	11	17	12		
M063	15	13	29	24	10	13	12	15,18	10	12	14	18	15	18	22	10	18	23	12	11	17	12	HD62	1
M064	15	13	29	24	10	13	14	12,19	10	12	14	18	15	17	21	10	17	24	13	10	19	10	HD63	1
M065	15	13	29	25	10	13	12	12,19	10	11	15	19	14	17	21	13	17	24	11	11	19	10	HD64	1
M066	15	13	29	25	11	13	12	12,18	10	12	14	19	15	16	23	11	18	24	12	11	17	10	HD65	1
M067	15	13	29	26	11	14	12	12,19	11	12	15	20	15	18	20	11	18	26	12	11	18	11	HD66	1
M068	15	13	29	26	11	14	12	12,19	11	13	15	20	15	18	20	11	20	26	12	11	18	11	HD67	1
M069	15	13	30	22	10	11	15	11,11	10	11	14	21	15	15	20	11	19	27	12	12	17	8	HD68	1
M070	15	13	30	23	10	11	15	11,11	10	11	14	21	15	15	21	11	19	27	12	13	15	8	HD69	1
M071	15	13	30	23	10	12	12	12,12	11	12	15	21	15	21	24	13	20	21	11	11	19	11	HD70	1
M072	15	13	30	24	10	13	12	14,17.2	10	11	15	19	14	16	21	9	19	23	14	11	17	10	HD71	1
M073	15	13	30	24	10	13	14	13,19	10	12	14	17	15	16	22	11	18	23	11	10	16	13	HD72	1

M074	15	14	30	23	10	13	15	12,20	10	13	14	18	15	17	23	11	17	22	15	10	18	12	HD73	1
M075	15	14	30	23	9	11	15	18,19	10	11	14	21	15	15	21	11	19	26	12	11	16	8	HD74	1
M076	15	14	30	24	10	13	14	12,17	10	11	14	18	15	15	21	12	18	24	13	11	19	11	HD75	1
M077	15	14	32	23	10	14	13	11,12	11	11	14	19	16	16	24	12	17	22	12	13	18	10	HD76	1
M078	16	12	28	24	11	14	12	13,16	11	12	14	20	14	16	20	11	19	23	11	11	16	11	HD77	1
M079	16	12	28	24	9	13	12	12,20	10	12	14	19	14	17	22	12	20	26	13	11	18	10	HD78	1
M080	16	12	28	25	10	13	12	12,19	10	12	14	19	15	17	20	12	19	26	13	11	18	10	HD79	1
M081	16	12	28	25	11	13	12	15,17	10	12	14	18	14	18	24	11	17	24	12	11	19	10	HD80	1
M082	16	12	28	26	10	13	12	12,18	10	12	14	18	14	18	21	12	20	26	13	11	17	10	HD81	1
M083	16	13	29	23	10	11	14	11,18	10	10	14	20	15	16	20	12	14	24	12	11	16	9	HD82	1
M084	16	13	29	23	11	13	14	13,20	10	11	14	18	15	19	25	10	17	23	13	10	16	12	HD83	1
M085	16	13	29	24	11	13	14	13,16	10	12	14	18	15	16	21	11	18	17	12	10	17	12	HD84	1
M086	16	13	29	25	10	13	12	12,19	10	13	14	18	14	17	22	12	19	25	13	11	17	10	HD85	1
M087	16	13	29	25	11	14	13	11,12	10	10	14	18	15	16	23	12	16	23	13	12	18	10	HD86	1
M088	16	13	29	25	11	15	13	13,18	10	12	14	18	16	17	21	11	17	23	12	10	18	12	HD87	1

M089	16	13	30	23	10	11	14	11,16	10	11	14	22	15	15	20	10	18	24	12	12	18	8	HD88	1
M090	16	13	30	23	10	11	14	11,17	10	11	14	22	15	15	20	10	18	24	12	12	18	8	HD89	1
M091	16	13	30	25	10	13	14	13.2,18	10	12	14	18	14	17	24	10	17	23	11	10	16	13	HD90	1
M092	16	13	30	25	10	13	14	12,18	10	13	14	18	15	17	22	11	19	24	12	10	17	12	HD91	1
M093	16	13	30	25	11	13	14	14,18	10	12	14	18	15	18	23	11	19	24	12	10	17	12	HD92	1
M094	16	14	29	21	10	14	12	11,11	10	12	15	19	16	19	20	13	18	23	13	11	19	10	HD93	1
M095	17	12	26	25	11	13	12	12,19	10	12	14	19	15	18	21	11	19	26	13	11	18	10	HD94	1
M096	17	12	26	25	11	13	12	12,19	10	12	14	19	15	18	21	11	19	26	13	11	18	10	HD95	1
M097	17	12	29	24	10	13	12	10,17	10	12	15	20	14	17	23	12	18	23	13	11	19	10	HD96	1
M098	17	12	29	25	10	13	12	12,19	10	11	14	18	13	18	22	12	19	23	12	13	18	10	HD97	1
M099	17	13	27	26	10	13	12	14,18	10	13	14	18	14	18	23	12	17	24	12	11	18	10	HD98	1
M100	17	13	30	24	10	13	12	12,20	10	12	14	20	15	16	22	11	17	25	12	11	18	10	HD99	1
M101	17	13	31	25	10	14	12	11,18	10	12	15	19	15	18	22	11	17	22	12	11	16	11	HD100	1
M102	18	12	27	22	10	13	12	11,19	10	11	14	19	15	17	21	13	18	23	12	10	18	10	HD101	1

Supplementary Table S3. The haplotype data of 23 Y-STRs included in PowerPlex Y23 amplification system in 109 Qiandongnan Miao individuals.

ID	DYS19	DYS389I	DYS389II	DYS390	DYS391	DYS392	DYS393	DYS385	DYS438	DYS439	DYS437	DYS448	DYS456	DYS458	DYS635	YGATAH4	DYS576	DYS481	DYS549	DYS533	DYS570	DYS643	Haplotype	n
M001	14	12	27	25	10	13	12	13,20	10	12	15	20	15	18	22	12	18	27	12	11	18	11	HD1	1
M002	14	12	28	23	10	14	12	13,20	11	11	14	20	15	17	20	11	17	23	13	11	21	11	HD2	1
M003	14	12	28	23	10	14	12	15,19	11	11	15	20	15	19	20	12	17	23	13	12	17	11	HD3	1
M004	14	12	28	23	10	14	13	10,13	10	10	14	21	15	16	21	14	16	22	12	13	18	11	HD4	1
M005	14	12	29	23	9	12	12	12,18	10	12	15	19	15	17	19	13	19	22	12	11	16	11	HD5	1
M006	14	12	29	24	10	11	13	15,15	11	11	15	19	14	16	19	12	18	25	13	12	18	10	HD6	1
M007	14	12	29	24	10	14	12	13,17	11	12	15	20	15	18	20	12	21	24	12	11	16	12	HD7	1
M008	14	12	30	24	10	11	13	15,15	11	11	14	19	14	16	19	12	19	25	12	12	20	10	HD8	1
M009	14	13	29	23	10	14	13	13,14	10	12	14	18	16	16	19	12	16	24	14	11	18	11	HD9	1
M010	14	13	29	23	10	14	13	14,15	10	12	14	18	16	16	19	12	17	24	14	11	18	11	HD10	1
M011	14	13	29	23	10	15	13	13,13	9	13	14	17	15	16	19	12	19	24	12	11	20	11	HD11	1
M012	14	13	29	24	9	14	14	15,20	12	11	14	19	17	17	22	11	19	24	12	12	21	10	HD12	1
M013	14	13	31	24	10	14	12	13,18	11	13	14	20	14	19	20	12	19	23	13	11	18	12	HD13	1

M014	14	14	30	23	10	14	13	13,14	10	11	14	18	16	16	19	12	17	24	14	11	18	12	HD14	1
M015	14	14	30	23	10	14	13	13,14	10	12	14	18	15	16	19	12	16	24	13	11	18	11	HD15	1
M016	14	14	30	23	10	14	13	13,14	10	13	14	18	16	16	19	12	17	24	13	11	18	11	HD16	1
M017	14	14	31	24	10	11	13	15,17	11	11	15	19	14	16	20	12	19	25	12	11	16	10	HD17	1
M018	14	14	31	24	10	11	13	15,16	11	11	15	19	14	16	20	12	19	26	12	11	16	10	HD18	1
M019	15	12	27	23	10	12	13	12,12	10	11	15	19	14	17	21	11	18	23	13	12	17	12	HD19	1
M020	15	12	28	24	10	13	12	12,21	10	11	14	19	14	18	22	12	17	27	12	11	18	10	HD20	1
M021	15	12	28	24	10	13	12	12,21	10	11	14	19	14	18	22	12	18	27	12	11	18	10	HD21	1
M022	15	12	28	24	10	13	12	12,21	10	11	14	19	14	19	21	12	17	27	12	11	18	10	HD22	1
M023	15	12	28	24	10	13	12	13,22	10	11	15	20	15	17	23	12	19	22	12	11	20	10	HD23	1
M024	15	12	28	26	10	13	12	13,13	10	12	14	18	15	19	24	11	18	24	12	12	17	10	HD24	1
M025	15	12	29	23	10	14	13	13,14	10	12	14	19	15	15	23	12	16	25	13	12	20	11	HD25	1
M026	15	12	29	23	10	14	13	12,14	11	13	14	19	15	15	20	12	16	25	12	12	20	11	HD26	1
M027	15	12	29	23	11	14	13	12,13	10	11	14	18	17	15	19	12	18	25	12	11	19	11	HD27	1
M028	15	12	29	24	10	13	15	14,16	10	11	14	18	16	18	23	10	17	23	11	10	16	10	HD28	1

M029	15	12	30	23	10	14	13	13,14	10	12	14	18	15	15	22	12	17	29	11	12	18	11	HD29	1
M030	15	12	31	22	10	14	13	12,13	10	11	14	19	15	13	21	12	16	25	12	12	20	12	HD30	1
M031	15	12	31	23	10	13	12	11,18	10	11	14	20	16	17	22	11	19	23	11	12	16	11	HD31	1
M032	15	13	29	23	10	13	14	12,19	10	14	14	18	15	17	24	11	19	23	14	10	19	11	HD32	1
M033	15	13	29	23	11	13	13	20,20	10	11	14	18	15	17	24	10	19	23	12	10	16	12	HD33	1
M034	15	13	29	24	10	13	14	13,18	10	12	14	18	15	19	25	11	20	24	11	10	16	13	HD34	1
M035	15	13	29	24	10	14	12	13,17	11	11	15	20	14	16	20	12	20	23	12	13	18	11	HD35	1
M036	15	13	29	24	11	13	14	13,18	10	12	14	18	15	18	22	11	18	23	11	10	16	12	HD36	1
M037	15	13	29	25	10	13	14	16,21	9	12	14	18	15	15	21	11	18	24	12	10	16	12	HD37	1
M038	15	13	30	23	10	11	15	11,12	10	11	14	21	17	15	22	11	19	27	12	12	16	8	HD38	1
M039	15	13	30	24	10	13	12	12,21	10	11	14	19	14	18	22	12	17	27	12	11	19	10	HD39	1
M040	15	13	30	24	11	13	14	14,19	11	11	14	18	16	17	20	10	17	23	12	10	17	12	HD40	1
M041	15	13	30	25	10	13	14	14,18	10	11	14	18	15	17	22	9	18	23	10	10	16	13	HD41	1
M042	15	13	30	25	11	13	13	13,18	10	12	14	18	15	17	21	11	20	24	12	10	17	12	HD42	1
M043	15	13	31	23	10	11	15	11,11	10	11	14	21	15	15	22	11	17	27	12	12	15	8	HD43	1

M044	15	13	31	23	10	11	15	11,11	10	11	14	21	15	15	22	11	18	27	12	12	15	8	HD44	1
M045	15	13	31	23	10	11	16	11,11	10	11	14	21	15	15	22	11	17	27	12	12	15	8	HD45	1
M046	15	13	32	23	10	11	15	11,11	10	10	14	21	15	15	22	11	18	27	12	12	15	8	HD46	1
M047	15	14	30	23	10	11	15	11,11	10	11	14	21	15	16	21	11	18	25	12	13	18	8	HD47	1
M048	15	14	30	23	10	14	13	11,12	11	11	14	19	15	15	23	12	17	22	12	12	18	10	HD48	1
M049	15	14	30	24	10	13	12	14,20	10	12	15	19	14	18	22	12	16	23	12	11	17	11	HD49	1
M050	15	14	30	24	11	14	12	13,20	11	11	15	20	15	18	21	12	19	23	12	11	17	11	HD50	1
M051	15	14	30	25	10	13	14	15,19	10	12	14	18	16	16	22	11	21	24	12	10	18	12	HD51	1
M052	15	14	30	25	11	14	14	13,19	10	12	14	18	16	16	25	11	17	24	11	10	16	12	HD52	1
M053	15	14	31	23	10	13	14	14,15	10	11	14	18	15	15	23	12	21	23	12	9	17	10	HD53	1
M054	15	14	31	23	10	13	14	14,15	10	11	14	18	15	16	23	12	20	23	12	9	17	10	HD54	1
M055	15	14	31	24	10	13	14	12,21	10	12	14	18	15	18	21	11	19	23	14	10	19	11	HD55	1
M056	15	14	31	24	11	13	14	14,19	11	11	14	18	16	16	20	10	17	23	12	10	17	12	HD56	1
M057	15	14	32	24	11	13	14	14,19	11	11	14	18	16	17	20	10	20	23	12	10	17	11	HD57	1
M058	16	12	27	23	10	14	12	13,20	10	11	15	22	15	17	21	12	17	23	12	11	18	11	HD58	1

M059	16	12	28	23	10	13	12	15,19	10	11	15	20	15	17	21	12	20	24	12	11	19	11	HD59	1
M060	16	12	28	24	10	13	12	13,22	10	11	15	20	15	17	23	12	19	22	12	11	20	10	HD60	1
M061	16	12	28	24	10	13	12	13,22	10	11	15	20	15	19	23	12	18	22	12	11	20	10	HD61	1
M062	16	12	28	24	10	13	12	13,22	10	11	15	20	16	19	23	11	18	21	12	11	20	10	HD62	1
M063	16	12	28	24	10	13	12	13,22	10	12	15	20	15	19	23	11	19	22	11	11	20	10	HD63	1
M064	16	12	29	23	11	14	13	13,13	10	11	14	18	16	18	19	12	18	25	12	11	18	12	HD64	1
M065	16	13	29	24	11	13	13	12,19	10	12	14	18	15	17	24	11	18	23	11	10	16	12	HD65	1
M066	16	13	29	25	10	13	14	14,18	10	11	14	18	15	17	23	10	17	23	11	10	17	13	HD66	1
M067	16	13	30	24	10	12	13	13,18	10	12	14	18	15	18	23	11	19	24	12	10	17	12	HD67	1
M068	16	13	30	24	10	13	13	15,18	10	11	14	18	15	17	22	10	18	24	11	10	16	13	HD68	1
M069	16	13	30	24	10	13	14	14,19	10	11	14	18	15	17	23	10	18	23	11	10	16	13	HD69	1
M070	16	13	30	25	10	13	13	14,18	10	11	14	18	15	17	23	10	18	23	11	10	19	13	HD70	1
M071	16	13	30	25	10	13	15	14,18	10	11	14	18	15	17	23	10	18	23	11	10	17	13	HD71	1
M072	16	13	30	25	10	13	15	14,18	10	12	14	18	15	17	23	10	18	23	11	10	17	13	HD72	1
M073	16	13	31	24	12	13	14	13,20	10	13	14	18	15	18	24	11	18	23	10	10	16	12	HD73	1

M074	16	13	31	25	10	13	14	14,18	10	11	14	18	15	17	23	10	18	22	11	11	16	13	HD74	1
M075	16	14	30	23	10	11	13	11,17	10	11	14	21	15	18	21	11	16	24	12	12	16	9	HD75	1
M076	16	14	30	24	11	13	14	13,18	10	13	14	18	14	16	22	10	19	23	12	10	18	10	HD76	1
M077	16	14	31	25	10	12	12	19,19	10	13	15	20	13	18	21	12	18	23	12	11	19	11	HD77	1
M078	17	11	27	24	10	13	12	13,21	11	11	14	20	15	20	23	12	19	22	12	11	20	10	HD78	1
M079	17	11	27	24	10	13	12	13,21	10	11	15	20	15	18	23	12	19	22	11	11	19	10	HD79	1
M080	17	11	27	24	10	13	12	13,21	10	11	15	20	15	19	22	12	18	22	12	11	19	10	HD80	1
M081	17	11	27	24	10	13	12	13,19	10	11	15	20	15	19	23	11	19	22	13	11	20	10	HD81	1
M082	17	11	27	24	10	13	12	13,21	10	11	15	20	15	19	23	12	17	22	12	12	23	10	HD82	1
M083	17	11	27	24	10	13	12	13,21	10	11	15	20	15	19	23	12	19	22	12	11	20	10	HD83	2
M084	17	11	27	24	10	13	12	13,21	10	11	15	20	15	19	23	12	19	22	12	11	20	10		
M085	17	11	27	24	10	13	13	13,21	10	11	14	20	15	19	23	12	20	22	12	11	20	10	HD84	1
M086	17	12	28	24	10	12	12	13,22	10	12	15	20	15	19	23	12	19	22	12	11	18	10	HD85	1
M087	17	12	28	24	10	13	12	13,22	10	11	15	20	15	19	22	12	18	21	12	11	19	8	HD86	1
M088	17	12	28	24	10	13	12	13,22	10	11	15	20	15	19	22	12	18	22	12	11	19	10	HD87	1

M089	17	12	28	24	10	13	12	13,22	10	11	15	20	15	19	22	12	18	22	12	11	20	10	HD88	1
M090	17	12	28	24	10	13	12	13,21	10	11	15	20	15	19	23	12	18	22	12	11	19	10	HD89	1
M091	17	12	28	24	10	13	12	13,22	10	11	15	20	15	19	23	12	19	22	12	11	20	10	HD90	1
M092	17	12	28	24	10	13	12	13,23	10	11	15	20	15	19	24	12	19	22	12	11	21	10	HD91	1
M093	17	12	28	24	10	13	12	13,23	10	11	15	20	16	19	23	12	19	22	12	11	19	10	HD92	1
M094	17	12	28	24	10	13	12	13,22	10	12	15	20	15	19	23	12	19	22	12	11	19	10	HD93	1
M095	17	12	28	24	10	13	12	13,22	10	12	15	20	15	19	23	12	19	22	12	11	20	10	HD94	1
M096	17	12	28	24	10	13	12	13,22	10	13	15	20	15	18	23	12	19	22	13	11	20	10	HD95	1
M097	17	12	28	24	10	13	13	13,21	10	11	15	20	15	19	23	12	19	23	12	11	20	10	HD96	1
M098	17	12	28	24	10	14	13	13,22	10	11	15	20	15	18	23	12	18	22	12	11	21	10	HD97	1
M099	17	12	29	24	10	13	12	13,21	10	10	14	20	15	17	23	12	17	22	13	11	20	10	HD98	1
M100	17	12	29	24	10	13	12	13,21	10	10	15	20	15	17	22	11	17	23	12	11	19	10	HD99	1
M101	17	12	29	24	10	13	12	13,20	10	10	15	20	15	17	23	12	16	22	12	11	20	10	HD100	1
M102	17	12	29	24	10	13	12	13,22	10	10	15	20	15	17	23	12	17	22	12	11	18	10	HD101	1
M103	17	12	29	24	10	13	12	13,21	10	10	15	20	15	17	23	12	17	22	12	11	20	10	HD102	1

M10 4	17	12	29	24	10	13	12	13,21	10	10	15	21	15	17	23	12	19	22	12	11	19	10	HD103	1
M10 5	17	12	29	24	10	13	12	13,20	10	11	15	20	15	17	23	12	17	22	12	11	19	10	HD104	1
M10 6	17	12	29	24	10	13	12	13,21	10	12	15	20	15	19	22	12	18	22	12	11	19	10	HD105	1
M10 7	17	12	29	24	10	13	12	13,22	10	13	15	20	16	19	23	11	18	22	12	11	20	10	HD106	1
M10 8	17	13	28	23	9	12	13	12,12	10	12	15	18	16	17	19	11	20	22	12	11	16	11	HD107	1
M10 9	17	14	29	24	10	14	12	14,21	12	12	15	20	15	17	23	11	17	25	13	11	20	12	HD108	1

Supplementary Table S4. The allele frequency and gene diversity of 23 Y-STR loci included in PowerPlex 23 in Bouyei residing in Qiannan Bouyei population in Guizhou Province (n=98).

Allele	DYS19	DYS389I	DYS389II	DYS390	DYS391	DYS392	DYS393	DYS437	DYS438	DYS439	DYS448	DYS456	DYS458	DYS635	Y GATA H4	DYS576	DYS481	DYS549	DYS533	DYS570	DYS643	Allele	DYS385a/b	Allele	DYS385a/b
9					0.0408				0.0204	0.0102									0.0102		0.0612	11,12	0.0204	14,16	0.0204
10		0.0102			0.7857	0.0102			0.6122	0.0408					0.0816				0.1531		0.4490	11,17	0.0102	14,17	0.0102
11		0.0102			0.1735	0.2245			0.3469	0.3776					0.3061			0.1122	0.5102		0.2347	11,18	0.0102	14,18	0.0612
12		0.5510				0.0204	0.4184		0.0204	0.4592					0.4592			0.5510	0.3163		0.1531	11,19	0.0306	14,21	0.0102
13	0.0204	0.3265				0.4592	0.3469	0.0030		0.1122			0.0204		0.1429			0.2551	0.0102	0.0102	0.1020	12,13	0.0102	15,15	0.1327
14	0.3367	0.1020				0.2755	0.2041	0.6518				0.2347	0.0204		0.0102			0.0714				12,14	0.0102	15,16	0.0204
15	0.3673					0.0102	0.0204	0.3363				0.5306	0.1224					0.0102		0.0204		12,16	0.0204	15,21	0.0102
16	0.2041						0.0102	0.0089			0.0102	0.2041	0.1837			0.0408				0.2041		12,17	0.0306	15,22	0.0102
17	0.0714											0.0204	0.1735			0.2041				0.2653		12,18	0.0102	16,16	0.0102
18											0.3367		0.2041			0.3265				0.1224		12,19	0.0204	16,17	0.0204
19											0.2755	0.0102	0.1531	0.1939		0.2857				0.1327		12,20	0.0204	16,18	0.0510
20											0.3571		0.0918	0.1837		0.1224				0.2245		12,21	0.0306	16,19	0.0102
21				0.0306							0.0102		0.0204	0.1531		0.0204	0.1735			0.0102		13,13	0.0204	17,18	0.0102
21.3														0.0102								13,14	0.0816		
22				0.0306							0.0102		0.0102	0.1735			0.1020			0.0102		13,16	0.0102		
23				0.1837										0.2143			0.1837					13,17	0.0510		
24				0.5816										0.0408			0.2041					13,18	0.0204		
25				0.1633										0.0306			0.2143					13,19	0.0306		
26			0.0102	0.0102													0.0612					13,20	0.0102		
27			0.0816														0.0408					13,21	0.0102		
28			0.3367														0.0204					13,22	0.1224		
29			0.1837																			13,23	0.0102		
30			0.3061																			13,24	0.0102		
31			0.0714																			14,14	0.0102		
32			0.0102																			14,15	0.0102		
GD	0.7118	0.5851	0.7549	0.6136	0.3545	0.6691	0.6693	0.4639	0.5092	0.6388	0.6899	0.6276	0.8550	0.8351	0.6752	0.7608	0.8409	0.6198	0.6223	0.8125	0.7130	-	-	-	0.9520

Supplementary Table S5. The allele frequency and forensic parameters of 23 Y-STRs included in PowerPlex 23 kit in Han individuals living in Zunyi, Guizhou Province (n=101).

Allele	DYS19	DYS389I	DYS389II	DYS390	DYS391	DYS392	DYS393	DYS437	DYS438	DYS439	DYS448	DYS456	DYS458	DYS635	Y GATA H4	DYS576	DYS481	DYS549	DYS533	DYS570	DYS643	Allele	DYS385a/b	Allele	DYS385a/b
7							0.0098															10,17	0.0098	14,17	0.0098
8																					0.0490	11,11	0.0294	14,17.2	0.0098
9					0.0588				0.0196						0.0098						0.0392	11,12	0.0490	14,18	0.0686
10					0.6961	0.0098			0.7451	0.0588					0.1373				0.1667		0.2647	11,14	0.0098	14,19	0.0098
11					0.2353	0.0882	0.0098		0.2157	0.3333					0.3529			0.1373	0.5882		0.4118	11,16	0.0098	14,20	0.0098
12		0.4804			0.0098	0.0980	0.5588		0.0196	0.4608					0.4510			0.5784	0.2059		0.2157	11,17	0.0098	15,17	0.0098
13	0.0490	0.4314				0.4412	0.1863	0.0029		0.1373		0.0294			0.0490			0.1961	0.0392		0.0196	11,18	0.0196	15,18	0.0196
14	0.1961	0.0882				0.3039	0.1863	0.7000		0.0098		0.1863				0.0098		0.0784		0.0098		11,19	0.0098	15,19	0.0098
15	0.5098					0.0392	0.0490	0.2882				0.6176	0.1569					0.0098		0.0098		12,12	0.0392	15,20	0.0196
16	0.1667					0.0196		0.0088				0.1176	0.1765			0.1078				0.1961		12,16	0.0490	16,17	0.0098
17	0.0686										0.0294	0.0294	0.2353	0.0098		0.2157	0.0196			0.1765		12,17	0.0588	18,19	0.0098
18	0.0098										0.3725	0.0196	0.3235			0.3235				0.2647		12,18	0.0294	13.2,18	0.0098
19											0.2843		0.0784	0.1078		0.1961				0.2353		12,19	0.0784		
20											0.2549		0.0196	0.2157		0.1275	0.0196			0.0686		12,20	0.0588		
21				0.0098							0.0392		0.0098	0.2647		0.0196	0.0588			0.0294		12,21	0.0098		
22				0.0392							0.0196			0.1863			0.0980			0.0098		12,22	0.0098		
23				0.3627										0.1078			0.3137					13,13	0.0392		
24				0.3137										0.0784			0.2059					13,14	0.0686		
25				0.2353										0.0294			0.1176					13,15	0.0098		
26			0.0098	0.0392													0.0980					13,16	0.0294		
27			0.0882														0.0490					13,17	0.0196		
28			0.2745														0.0196					13,18	0.0098		
29			0.3725																			13,19	0.0784		
30			0.2353																			13,20	0.0196		
31			0.0098																			13,21	0.0196		
32			0.0098																			14,14	0.0294		
GD：	0.6733	0.5811	0.7296	0.7185	0.4611	0.7004	0.6218	0.4286	0.4015	0.6606	0.7197	0.5735	0.7853	0.8264	0.6572	0.7897	0.8272	0.6078	0.5880	0.8070	0.7166	-	-	-	0.9654

Supplementary Table S6. The allele frequency and gene diversity of 23 Y-STRs included in PowerPlex Y23.

Allele	DYS19	DYS389I	DYS389II	DYS390	DYS391	DYS392	DYS393	DYS437	DYS438	DYS439	DYS448	DYS456	DYS458	DYS635	Y GATA H4	DYS576	DYS481	DYS549	DYS533	DYS570	DYS643	Allele	DYS385a/b	Allele	DYS385a/b
8																					0.0642	10,13	0.0092	14,21	0.0092
9					0.0275				0.0183						0.0092				0.0183		0.0092	11,11	0.0459	15,15	0.0183
10					0.8532				0.8165	0.0734					0.1193			0.0183	0.2202		0.4495	11,12	0.0183	15,16	0.0092
11		0.0734			0.1101	0.1009			0.1468	0.5872					0.2752			0.1468	0.5596		0.2294	11,17	0.0092	15,17	0.0092
12		0.4587			0.0092	0.0550	0.4679		0.0183	0.2477					0.5780			0.6789	0.1743		0.1651	11,18	0.0092	15,18	0.0092
13		0.2844				0.6147	0.2752	0.0029		0.0826		0.0092	0.0092		0.0092			0.1101	0.0275		0.0826	12,12	0.0183	15,19	0.0275
14	0.1651	0.1835				0.2202	0.1743	0.6542		0.0092		0.1193			0.0092			0.0459				12,13	0.0183	15,20	0.0092
15	0.3578					0.0092	0.0734	0.3343				0.6972	0.1101							0.0367		12,14	0.0092	16,21	0.0092
16	0.1835						0.0092	0.0086				0.1468	0.1651			0.0826				0.1835		12,18	0.0092	19,19	0.0092
17	0.2936										0.0092	0.0275	0.2936			0.2294				0.1376		12,19	0.0183	20,20	0.0092
18											0.3394		0.1651			0.2936				0.2018		12,21	0.0459		
19											0.1468		0.2477	0.1101		0.2936				0.1651		13,13	0.0275		
20											0.4128		0.0092	0.1009		0.0734				0.2294		13,14	0.0550		
21											0.0826			0.1193		0.0275	0.0183			0.0367		13,17	0.0183		
22				0.0092							0.0092			0.2202			0.3303					13,18	0.0550		
23				0.2936										0.3761			0.2844			0.0092		13,19	0.0183		
24				0.5780										0.0550			0.1651					13,20	0.0642		
25				0.1101										0.0183			0.0917					13,21	0.1284		
26				0.0092													0.0092					13,22	0.1468		
27			0.1009														0.0917					13,23	0.0183		
28			0.2477																			14,15	0.0275		
29			0.2752														0.0092					14,16	0.0092		
30			0.2202																			14,18	0.0550		
31			0.1376																			14,19	0.0367		
32			0.0183																			14,20	0.0092		
GD	0.7316	0.6758	0.7922	0.5806	0.2615	0.5656	0.6757	0.4622	0.3140	0.5870	0.6922	0.4817	0.7929	0.7773	0.5810	0.7691	0.7725	0.5076	0.6125	0.8316	0.7136	-	-	-	0.9468

Supplementary Table S7. The pairwise Rst genetic distances between the three studied populations and 8 reference populations (Han Chinese populations).

Population	[01]	[02]	[03]	[04]	[05]	[06]	[07]	[08]	[09]	[10]	[11]
[01]Zunyi-Han											
[02]Qiandongnan-Miao	0.0429										
[03]Qiannan-Bouyei	0.0153	0.0171									
[04]Beijing-Han	0.0037	0.0526	0.0211								
[05]China-Han	0.0083	0.0657	0.0294	0.0092							
[06]Hunan-Han	0.0013	0.0548	0.0221	0.0061	0.0158						
[07]Jiangsu-Han	0.0041	0.0677	0.0297	-0.0001	0.0113	0.0056					
[08]Shanghai-Han	0.0071	0.0777	0.0382	0.0070	0.0000	0.0094	0.0045				
[09]Southern-Han	0.0327	0.0175	0.0282	0.0340	0.0580	0.0369	0.0494	0.0606			
[10]Xuanwei-Han	0.0011	0.0374	0.0104	0.0047	0.0164	0.0045	0.0096	0.0167	0.0162		
[11]Minnan-Han	0.0282	0.0732	0.0229	0.0182	0.0290	0.0209	0.0242	0.0302	0.0562	0.0165	

Supplementary Table S8. The pairwise Rst genetic distances between the three studied populations and 19 reference populations (Chinese minorities).

Population	[01]	[02]	[03]	[04]	[05]	[06]	[07]	[08]	[09]	[10]	[11]	[12]	[13]	[14]	[15]	[16]	[17]	[18]	[19]	[20]	[21]	[22]
[01]Zunyi-Han																						
[02]Qiandongnan-Miao	0.0429																					
[03]Qiannan-Bouyei	0.0153	0.0171																				
[04]Yunnan-Bai	0.0281	0.0846	0.0439																			
[05]Xishuangbanna-Dai	0.0391	0.0797	0.0295	0.0260																		
[06]Guizhou-Gelao	0.0044	0.0560	0.0250	0.0442	0.0512																	
[07]Meizhou-Hakka Han	0.0345	0.1250	0.0807	0.0722	0.0942	0.0346																
[08]Cangzhou-Hui	0.0428	0.1114	0.0796	0.0696	0.1212	0.0398	0.0356															
[09]Gansu-Hui	0.0088	0.0460	0.0227	0.0482	0.0750	0.0175	0.0602	0.0325														
[10]Ningxia-Hui	0.0255	0.0906	0.0590	0.0418	0.0996	0.0239	0.0300	0.0026	0.0225													
[11]Qinghai-Hui	0.0443	0.1091	0.0740	0.0789	0.1352	0.0411	0.0429	0.0101	0.0295	0.0016												
[12]Chengde-Manchu	0.0030	0.0547	0.0236	0.0236	0.0401	0.0087	0.0399	0.0353	0.0082	0.0213	0.0389											
[13]Liaoning-Manchu	0.0057	0.0596	0.0270	0.0290	0.0463	0.0067	0.0414	0.0338	0.0065	0.0215	0.0384	-0.0007										
[14]Zunyi-Miao	0.0133	0.0719	0.0339	0.0569	0.0702	-0.0006	0.0304	0.0347	0.0195	0.0197	0.0278	0.0170	0.0122									
[15]Inner-Mongolia-Mongolian	0.0542	0.1387	0.1021	0.0930	0.1420	0.0533	0.0492	0.0253	0.0449	0.0166	0.0031	0.0535	0.0508	0.0430								
[16]Ordos-Western-Mongolian	0.0462	0.1242	0.0849	0.0837	0.1289	0.0377	0.0395	0.0161	0.0340	0.0089	-0.0002	0.0420	0.0373	0.0234	0.0045							
17]Fujian-She	0.0310	0.0210	0.0290	0.0658	0.0798	0.0453	0.0919	0.0861	0.0306	0.0742	0.0881	0.0320	0.0323	0.0561	0.1076	0.0936						
[18]Guizhou-Shui	0.0695	0.0446	0.0691	0.1385	0.1545	0.0748	0.1210	0.0909	0.0597	0.0836	0.0879	0.0846	0.0815	0.0786	0.1109	0.0992	0.0648					
[19]Tibet-Tibetan	0.1556	0.2263	0.1747	0.1952	0.2344	0.1540	0.1689	0.1441	0.1285	0.1165	0.0845	0.1632	0.1575	0.1169	0.1020	0.0802	0.1974	0.2014				
[20]Xinjiang-Uighur	0.0710	0.1412	0.1089	0.0980	0.1717	0.0651	0.0525	0.0132	0.0536	0.0087	0.0034	0.0629	0.0633	0.0518	0.0188	0.0112	0.1261	0.1125	0.0983			
[21]Liangshan-Yi	0.0155	0.0575	0.0154	0.0505	0.0495	0.0225	0.0677	0.0613	0.0184	0.0431	0.0501	0.0217	0.0258	0.0258	0.0752	0.0596	0.0584	0.0895	0.1461	0.0790		
[22]Guangxi-Zhuang	0.0401	0.0626	0.0315	0.0731	0.0588	0.0363	0.0997	0.1089	0.0688	0.0810	0.1132	0.0588	0.0628	0.0523	0.1250	0.1121	0.1061	0.1089	0.2215	0.1402	0.0431	

Supplementary Table S9. The pairwise Rst genetic distances between the three studied populations and 27 Chinese reference populations (mainland Chinese populations).

Population	[01]	[02]	[03]	[04]	[05]	[06]	[07]	[08]	[09]	[10]	[11]	[12]	[13]	[14]	[15]	[16]	[17]	[18]	[19]	[20]	[21]	[22]	[23]	[24]	[25]	[26]	[27]	[28]	[29]	[30]
[01]Zunyi-Han																														
[02]Qiandongnan-Miao	0.0429																													
[03]Qiannan-Bouyei	0.0153	0.0171																												
[04]Yunnan-Bai	0.0281	0.0846	0.0439																											
[05]Yunnan-Dai	0.0391	0.0797	0.0295	0.0260																										
[06]Guizhou-Gelao	0.0044	0.0560	0.0250	0.0442	0.0512																									
[07]Meizhou-Hakka Han	0.0345	0.1250	0.0807	0.0722	0.0942	0.0346																								
[08]Beijing-Han	0.0037	0.0526	0.0211	0.0234	0.0378	0.0063	0.0410																							
[09]China-Han	0.0083	0.0657	0.0294	0.0430	0.0637	0.0144	0.0394	0.0092																						
[10]Hunan-Han	0.0013	0.0548	0.0221	0.0410	0.0367	0.0036	0.0326	0.0061	0.0158																					
[11]Jiangsu-Han	0.0041	0.0677	0.0297	0.0257	0.0417	0.0071	0.0327	-0.0001	0.0113	0.0056																				
[12]Shanghai-Han	0.0071	0.0777	0.0382	0.0314	0.0472	0.0089	0.0289	0.0070	0.0000	0.0094	0.0045																			
[13]Southern-Han	0.0327	0.0175	0.0282	0.0850	0.0868	0.0382	0.1195	0.0340	0.0580	0.0369	0.0494	0.0606																		
[14]Xuanwei-Han	0.0011	0.0374	0.0104	0.0338	0.0272	0.0111	0.0537	0.0047	0.0164	0.0045	0.0096	0.0167	0.0162																	
[15]Cangzhou-Hui	0.0428	0.1114	0.0796	0.0696	0.1212	0.0398	0.0356	0.0406	0.0459	0.0581	0.0400	0.0406	0.1170	0.0648																
[16]Gansu-Hui	0.0088	0.0460	0.0227	0.0482	0.0750	0.0175	0.0602	0.0087	0.0127	0.0238	0.0151	0.0222	0.0347	0.0154	0.0325															
[17]Ningxia-Hui	0.0255	0.0906	0.0590	0.0418	0.0996	0.0239	0.0300	0.0241	0.0295	0.0397	0.0255	0.0264	0.0910	0.0472	0.0026	0.0225														
[18]Qinghai-Hui	0.0443	0.1091	0.0740	0.0789	0.1352	0.0411	0.0429	0.0435	0.0352	0.0600	0.0467	0.0462	0.1125	0.0677	0.0101	0.0295	0.0016													
[19]Chengde-Manchu	0.0030	0.0547	0.0236	0.0236	0.0401	0.0087	0.0399	-0.0008	0.0136	0.0091	0.0026	0.0107	0.0406	0.0079	0.0353	0.0082	0.0213	0.0389												
[20]Liaoning-Manchu	0.0057	0.0596	0.0270	0.0290	0.0463	0.0067	0.0414	-0.0013	0.0125	0.0102	0.0016	0.0095	0.0397	0.0086	0.0338	0.0065	0.0215	0.0384	-0.0007											
[21]Zunyi-Miao	0.0133	0.0719	0.0339	0.0569	0.0702	-0.0006	0.0304	0.0143	0.0178	0.0101	0.0130	0.0156	0.0540	0.0242	0.0347	0.0195	0.0197	0.0278	0.0170	0.0122										
[22]Minnan-Han	0.0282	0.0732	0.0229	0.0430	0.0151	0.0317	0.0756	0.0182	0.0290	0.0209	0.0242	0.0302	0.0562	0.0165	0.1018	0.0492	0.0796	0.1005	0.0265	0.0272	0.0420									
[23]inner Mongolia-Mongolian	0.0542	0.1387	0.1021	0.0930	0.1420	0.0533	0.0492	0.0568	0.0481	0.0721	0.0579	0.0556	0.1270	0.0812	0.0253	0.0449	0.0166	0.0031	0.0535	0.0508	0.0430	0.1104								
[24]Ordos-Western-Mongolian	0.0462	0.1242	0.0849	0.0837	0.1289	0.0377	0.0395	0.0444	0.0394	0.0579	0.0459	0.0461	0.1082	0.0675	0.0161	0.0340	0.0089	-0.0002	0.0420	0.0373	0.0234	0.0976	0.0045							
[25]Fujian-She	0.0310	0.0210	0.0290	0.0658	0.0798	0.0453	0.0919	0.0278	0.0434	0.0384	0.0359	0.0486	0.0386	0.0245	0.0861	0.0306	0.0742	0.0881	0.0320	0.0323	0.0561	0.0586	0.1076	0.0936						
[26]Guizhou-Shui	0.0695	0.0446	0.0691	0.1385	0.1545	0.0748	0.1210	0.0808	0.0819	0.0849	0.0937	0.0986	0.0488	0.0733	0.0909	0.0597	0.0836	0.0879	0.0846	0.0815	0.0786	0.1223	0.1109	0.0992	0.0648					
[27]Tibet-Tibetan	0.1556	0.2263	0.1747	0.1952	0.2344	0.1540	0.1689	0.1626	0.1374	0.1735	0.1759	0.1782	0.2067	0.1805	0.1441	0.1285	0.1165	0.0845	0.1632	0.1575	0.1169	0.2033	0.1020	0.0802	0.1974	0.2014				
[28]Xinjiang-Uighur	0.0710	0.1412	0.1089	0.0980	0.1717	0.0651	0.0525	0.0690	0.0626	0.0884	0.0733	0.0683	0.1450	0.0966	0.0132	0.0536	0.0087	0.0034	0.0629	0.0633	0.0518	0.1437	0.0188	0.0112	0.1261	0.1125	0.0983			
[29]Liangshan-Yi	0.0155	0.0575	0.0154	0.0505	0.0495	0.0225	0.0677	0.0242	0.0199	0.0237	0.0281	0.0292	0.0562	0.0195	0.0613	0.0184	0.0431	0.0501	0.0217	0.0258	0.0258	0.0393	0.0752	0.0596	0.0584	0.0895	0.1461	0.0790		
[30]Guangxi-Zhuang	0.0401	0.0626	0.0315	0.0731	0.0588	0.0363	0.0997	0.0581	0.0694	0.0441	0.0601	0.0576	0.0621	0.0447	0.1089	0.0688	0.0810	0.1132	0.0588	0.0628	0.0523	0.0670	0.1250	0.1121	0.1061	0.1089	0.2215	0.1402	0.0431	

Supplementary Table S10. The pairwise Rst genetic distances between the three studied populations and 58 Chinese reference populations (Asian populations).

Population	[01]	[02]	[03]	[04]	[05]	[06]	[07]	[08]	[09]	[10]	[11]	[12]	[13]	[14]	[15]	[16]	[17]	[18]	[19]	[20]	[21]	[22]	[23]	[24]	[25]	[26]	[27]	[28]	[29]	[30]
[01]Zunyi-Han																														
[02]Qiandongnan-Miao	0.0429																													
[03]Qiannan-Bouyei	0.0153	0.0171																												
[04]Yunnan-Bai	0.0281	0.0846	0.0439																											
[05]Xishuangbanna-Dai	0.0391	0.0797	0.0295	0.0260																										
[06]Guizhou-Gelao	0.0044	0.0560	0.0250	0.0442	0.0512																									
[07]Meizhou-HakkaHan	0.0345	0.1250	0.0807	0.0722	0.0942	0.0346																								
[08]Beijing-Han	0.0037	0.0526	0.0211	0.0234	0.0378	0.0063	0.0410																							
[09]China-Han	0.0083	0.0657	0.0294	0.0430	0.0637	0.0144	0.0394	0.0092																						
[10]Hunan-Han	0.0013	0.0548	0.0221	0.0410	0.0367	0.0036	0.0326	0.0061	0.0158																					
[11]Jiangsu-Han	0.0041	0.0677	0.0297	0.0257	0.0417	0.0071	0.0327	-0.0001	0.0113	0.0056																				
[12]Shangha-Han	0.0051	0.0751	0.0367	0.0318	0.0523	0.0042	0.0248	0.0026	0.0104	0.0058	0.0003																			
[13]SouthernChina-Han	0.0327	0.0175	0.0282	0.0850	0.0868	0.0382	0.1195	0.0340	0.0580	0.0369	0.0494	0.0559																		
[14]Xuanwei-Han	-0.0003	0.0471	0.0132	0.0287	0.0248	0.0131	0.0440	0.0066	0.0141	0.0033	0.0086	0.0128	0.0319																	
[15]Cangzhou-Hui	0.0428	0.1114	0.0796	0.0696	0.1212	0.0398	0.0356	0.0406	0.0459	0.0581	0.0400	0.0339	0.1170	0.0643																
[16]Gansu-Hui	0.0088	0.0460	0.0227	0.0482	0.0750	0.0175	0.0602	0.0087	0.0127	0.0238	0.0151	0.0186	0.0347	0.0211	0.0325															
[17]Ningxia-Hui	0.0255	0.0906	0.0590	0.0418	0.0996	0.0239	0.0300	0.0241	0.0295	0.0397	0.0255	0.0189	0.0910	0.0439	0.0026	0.0225														
[18]Qinghai-Hui	0.0443	0.1091	0.0740	0.0789	0.1352	0.0411	0.0429	0.0435	0.0352	0.0600	0.0467	0.0379	0.1125	0.0625	0.0101	0.0295	0.0016													
[19]Chengde-Manchu	0.0029	0.0535	0.0229	0.0231	0.0392	0.0083	0.0394	-0.0010	0.0130	0.0087	0.0024	0.0048	0.0393	0.0088	0.0351	0.0081	0.0208	0.0381												
[20]Liaoning-[Manchu	0.0057	0.0596	0.0270	0.0290	0.0463	0.0067	0.0414	-0.0013	0.0125	0.0102	0.0016	0.0037	0.0397	0.0113	0.0338	0.0065	0.0215	0.0384	-0.0007											
[21]Zunyi-Miao	0.0133	0.0719	0.0339	0.0569	0.0702	-0.0006	0.0304	0.0143	0.0178	0.0101	0.0130	0.0078	0.0540	0.0242	0.0347	0.0195	0.0197	0.0278	0.0163	0.0122										
[22]Minnan-MinHan	0.0282	0.0732	0.0229	0.0430	0.0151	0.0317	0.0756	0.0182	0.0290	0.0209	0.0242	0.0305	0.0562	0.0163	0.1018	0.0492	0.0796	0.1005	0.0258	0.0272	0.0420									
[23]InnerMongolia-Mongolian	0.0542	0.1387	0.1021	0.0930	0.1420	0.0533	0.0492	0.0568	0.0481	0.0721	0.0579	0.0470	0.1270	0.0758	0.0253	0.0449	0.0166	0.0031	0.0531	0.0508	0.0430	0.1104								
[24]Ordos-Western-Mongolian	0.0462	0.1242	0.0849	0.0837	0.1289	0.0377	0.0395	0.0444	0.0394	0.0579	0.0459	0.0358	0.1082	0.0636	0.0161	0.0340	0.0089	-0.0002	0.0413	0.0373	0.0234	0.0976	0.0045							
[25]Fujian-She	0.0310	0.0210	0.0290	0.0658	0.0798	0.0453	0.0919	0.0278	0.0434	0.0384	0.0359	0.0459	0.0386	0.0306	0.0861	0.0306	0.0742	0.0881	0.0308	0.0323	0.0561	0.0586	0.1076	0.0936						
[26]Guizhou-Shui	0.0695	0.0446	0.0691	0.1385	0.1545	0.0748	0.1210	0.0808	0.0819	0.0849	0.0937	0.0908	0.0488	0.0817	0.0909	0.0597	0.0836	0.0879	0.0832	0.0815	0.0786	0.1223	0.1109	0.0992	0.0648					
[27]Tibet-Tibetan	0.1556	0.2263	0.1747	0.1952	0.2344	0.1540	0.1689	0.1626	0.1374	0.1735	0.1759	0.1627	0.2067	0.1721	0.1441	0.1285	0.1165	0.0845	0.1614	0.1575	0.1169	0.2033	0.1020	0.0802	0.1974	0.2014				
[28]Xinjiang-Uighur	0.0710	0.1412	0.1089	0.0980	0.1717	0.0651	0.0525	0.0690	0.0626	0.0884	0.0733	0.0617	0.1450	0.0931	0.0132	0.0536	0.0087	0.0034	0.0618	0.0633	0.0518	0.1437	0.0188	0.0112	0.1261	0.1125	0.0983			
[29]Liangshan-Yi	0.0155	0.0575	0.0154	0.0505	0.0495	0.0225	0.0677	0.0242	0.0199	0.0237	0.0281	0.0301	0.0562	0.0172	0.0613	0.0184	0.0431	0.0501	0.0212	0.0258	0.0258	0.0393	0.0752	0.0596	0.0584	0.0895	0.1461	0.0790		
[30]Guangxi-Zhuang	0.0401	0.0626	0.0315	0.0731	0.0588	0.0363	0.0997	0.0581	0.0694	0.0441	0.0601	0.0594	0.0621	0.0473	0.1089	0.0688	0.0810	0.1132	0.0580	0.0628	0.0523	0.0670	0.1250	0.1121	0.1061	0.1089	0.2215	0.1402	0.0431	
[31]Chhattisgarh-India-Muria	0.0961	0.1094	0.0779	0.1278	0.1849	0.0798	0.1284	0.0934	0.0917	0.1078	0.1009	0.0938	0.1540	0.1112	0.0809	0.0863	0.0608	0.0577	0.0888	0.0944	0.0715	0.1430	0.0977	0.0779	0.1377	0.1017	0.1409	0.0845	0.0633	0.1082
[32]Southern-India-India-Tamil	0.0925	0.1398	0.1138	0.1186	0.1971	0.0783	0.0866	0.0853	0.0895	0.1076	0.0909	0.0799	0.1665	0.1189	0.0279	0.0694	0.0221	0.0214	0.0786	0.0829	0.0676	0.1641	0.0493	0.0388	0.1435	0.1125	0.1271	0.0098	0.0861	0.1401
[33]Andhra-Pradesh-India-Thoti	0.1250	0.1781	0.1579	0.1627	0.3219	0.0993	0.1085	0.1243	0.1151	0.1421	0.1303	0.1074	0.2541	0.1617	0.0502	0.1116	0.0071	-0.0129	0.1136	0.1242	0.0669	0.2502	0.0189	0.0150	0.2021	0.1387	0.0535	-0.0142	0.1147	0.1807

[34]Gunma-Japan-Japanese	0.1049	0.1224	0.1078	0.1432	0.1792	0.0954	0.1464	0.1059	0.1274	0.0955	0.1117	0.1119	0.0933	0.1092	0.1656	0.1147	0.1282	0.1424	0.1009	0.1082	0.0908	0.1622	0.1645	0.1292	0.1413	0.1446	0.1468	0.1581	0.1115	0.1278
[35]Shizuoka-Japan-Japanese	0.0940	0.1232	0.1016	0.1228	0.1493	0.0948	0.1402	0.0978	0.1083	0.0922	0.1024	0.1038	0.0783	0.0944	0.1555	0.1017	0.1247	0.1364	0.0922	0.0964	0.0910	0.1396	0.1547	0.1237	0.1294	0.1366	0.1590	0.1531	0.1033	0.1279
[36]Tokyo-Japan-Japanese	0.0905	0.1039	0.0840	0.1131	0.1304	0.0929	0.1502	0.0951	0.1119	0.0908	0.1028	0.1073	0.0639	0.0915	0.1605	0.0976	0.1282	0.1478	0.0911	0.0959	0.0919	0.1313	0.1700	0.1365	0.1202	0.1357	0.1707	0.1639	0.1004	0.1104
[37]Tsukuba-Japan-Japanese	0.0681	0.0924	0.0732	0.0877	0.1037	0.0739	0.1193	0.0717	0.0849	0.0701	0.0776	0.0804	0.0493	0.0691	0.1318	0.0769	0.1031	0.1240	0.0694	0.0734	0.0794	0.1023	0.1404	0.1139	0.0970	0.1173	0.1683	0.1366	0.0832	0.0939
[38]Daejeon-South-Korea-Korean	0.0605	0.0850	0.0746	0.0597	0.0820	0.0706	0.1203	0.0514	0.0818	0.0625	0.0594	0.0676	0.0404	0.0541	0.1247	0.0696	0.1017	0.1305	0.0493	0.0535	0.0899	0.0854	0.1392	0.1206	0.0747	0.1241	0.2129	0.1464	0.0835	0.1091
[39]Seoul-South-Korea-Korean	0.0584	0.0791	0.0750	0.0615	0.0778	0.0731	0.1200	0.0493	0.0760	0.0642	0.0587	0.0676	0.0344	0.0551	0.1195	0.0627	0.1006	0.1300	0.0470	0.0516	0.0980	0.0791	0.1376	0.1250	0.0666	0.1196	0.2365	0.1445	0.0839	0.1098
[40]Ulaanbaatar-Mongolia-Mongolian	0.1718	0.2587	0.2252	0.2010	0.2812	0.1552	0.1420	0.1651	0.1627	0.1907	0.1673	0.1510	0.2589	0.1983	0.0865	0.1480	0.0833	0.0552	0.1565	0.1534	0.1353	0.2454	0.0393	0.0521	0.2303	0.2092	0.1447	0.0609	0.1883	0.2432
[41]Singapore-Han	0.0091	0.0595	0.0149	0.0282	0.0130	0.0141	0.0544	0.0058	0.0206	0.0034	0.0090	0.0138	0.0476	0.0033	0.0809	0.0330	0.0558	0.0793	0.0100	0.0132	0.0235	-0.0013	0.0922	0.0775	0.0441	0.1110	0.1819	0.1167	0.0247	0.0492
[42]Singapore-Indian	0.0842	0.1219	0.0980	0.1010	0.1687	0.0725	0.0923	0.0760	0.0860	0.0971	0.0857	0.0756	0.1397	0.1050	0.0396	0.0709	0.0256	0.0356	0.0704	0.0778	0.0662	0.1368	0.0692	0.0559	0.1371	0.0980	0.1592	0.0328	0.0748	0.1207
[43]Singapore-Malay	0.0341	0.0853	0.0545	0.0773	0.0972	0.0278	0.0432	0.0513	0.0586	0.0302	0.0479	0.0389	0.0943	0.0444	0.0730	0.0694	0.0413	0.0621	0.0505	0.0580	0.0260	0.0858	0.0844	0.0659	0.0969	0.0936	0.1510	0.0804	0.0472	0.0442
[44]Taiwan-Ami	0.1427	0.2317	0.1882	0.1943	0.2490	0.1121	0.1139	0.1475	0.1374	0.1284	0.1371	0.1177	0.2609	0.1547	0.1523	0.1865	0.1265	0.1281	0.1476	0.1527	0.0983	0.1984	0.1171	0.1170	0.2379	0.1910	0.2095	0.1594	0.1555	0.1636
[45]Taiwan-Atayal	0.3238	0.4022	0.3703	0.3700	0.4700	0.2789	0.2167	0.3213	0.3481	0.2905	0.2965	0.2773	0.5255	0.3204	0.2806	0.3787	0.2727	0.2649	0.3078	0.3184	0.2560	0.4253	0.2518	0.2370	0.4096	0.3465	0.2692	0.2752	0.3105	0.3589
[46]Taiwan-Bunun	0.3106	0.3707	0.3282	0.3898	0.4434	0.2776	0.2729	0.3159	0.3013	0.2848	0.3123	0.2921	0.4113	0.2973	0.3416	0.3585	0.3114	0.2831	0.3133	0.3245	0.2630	0.3667	0.2781	0.2638	0.3892	0.3242	0.2734	0.3252	0.2935	0.3403
[47]Taiwan-Paiwan	0.2557	0.3466	0.3038	0.3192	0.3987	0.2205	0.1945	0.2590	0.2624	0.2283	0.2475	0.2259	0.4015	0.2438	0.2706	0.3102	0.2316	0.2168	0.2578	0.2641	0.1930	0.3272	0.2114	0.1963	0.3404	0.2959	0.2257	0.2524	0.2581	0.3106
[48]Taiwan-Puyuma	0.1600	0.2540	0.2064	0.1981	0.2856	0.1466	0.1004	0.1740	0.1592	0.1469	0.1612	0.1420	0.3075	0.1574	0.1756	0.2208	0.1351	0.1359	0.1712	0.1812	0.1235	0.2381	0.1541	0.1355	0.2389	0.2310	0.1836	0.1589	0.1770	0.2291
[49]Taiwan-Rukai	0.2700	0.3714	0.3239	0.3170	0.4060	0.2339	0.1810	0.2720	0.2755	0.2429	0.2570	0.2333	0.4338	0.2650	0.2606	0.3285	0.2289	0.2206	0.2684	0.2758	0.2048	0.3460	0.2151	0.2012	0.3632	0.3262	0.2441	0.2443	0.2801	0.3280
[50]Taiwan-Saisiat	0.1324	0.2361	0.1761	0.1472	0.2229	0.1223	0.1086	0.1342	0.1148	0.1182	0.1180	0.1085	0.2907	0.1219	0.1695	0.1826	0.1310	0.1274	0.1340	0.1404	0.1081	0.1812	0.1316	0.1213	0.1990	0.2489	0.1707	0.1726	0.1352	0.2030
[51]Taiwan-Tsou	0.4026	0.4683	0.4485	0.4581	0.5633	0.3496	0.3094	0.3923	0.4353	0.3643	0.3751	0.3528	0.5948	0.3973	0.3767	0.4569	0.3518	0.3365	0.3814	0.3934	0.3309	0.4974	0.3141	0.3036	0.4806	0.4116	0.3098	0.3595	0.3940	0.4376
[52]Taiwan-Yami	0.2447	0.3401	0.2939	0.2537	0.3690	0.2272	0.1797	0.2489	0.2545	0.2308	0.2280	0.2154	0.4371	0.2366	0.2416	0.2985	0.2072	0.2052	0.2401	0.2490	0.2000	0.3337	0.2071	0.1919	0.3128	0.3267	0.2258	0.2377	0.2536	0.3143
[53]Northern-Thailand-Thailand-Karen-Padong	0.1508	0.0852	0.0679	0.1764	0.1473	0.1371	0.2622	0.1519	0.1733	0.1457	0.1723	0.1813	0.1207	0.1328	0.2616	0.1672	0.2122	0.2233	0.1529	0.1610	0.1387	0.1348	0.2622	0.2313	0.1615	0.1851	0.2767	0.2821	0.1143	0.0978
[54]Northern-Thailand-Thailand-Karen-Pwo	0.0789	0.0765	0.0487	0.0997	0.0699	0.0619	0.1393	0.0821	0.1065	0.0675	0.0946	0.0986	0.1221	0.0686	0.1537	0.1107	0.1079	0.1273	0.0814	0.0909	0.0669	0.0867	0.1622	0.1380	0.1199	0.1541	0.2154	0.1744	0.0659	0.0457
[55]Northern-Thailand-Thailand-Karen-Skaw	0.0626	0.0544	0.0356	0.0771	0.0514	0.0526	0.1152	0.0670	0.0899	0.0520	0.0776	0.0826	0.0938	0.0532	0.1339	0.0974	0.0957	0.1203	0.0644	0.0764	0.0604	0.0766	0.1606	0.1323	0.0891	0.1344	0.2195	0.1564	0.0570	0.0466
[56]Northern-Thailand-Thailand-Lawa	0.1890	0.1466	0.1245	0.2125	0.2150	0.1828	0.2788	0.2116	0.2618	0.1918	0.2239	0.2283	0.2244	0.1809	0.2771	0.2262	0.2323	0.2562	0.2008	0.2147	0.1815	0.2381	0.2901	0.2599	0.2514	0.2195	0.2989	0.2979	0.1602	0.1128
[57]Northern-Thailand-Thailand-Lua	0.3492	0.2582	0.2808	0.3643	0.3999	0.3118	0.4120	0.3424	0.4116	0.3343	0.3531	0.3568	0.3693	0.3306	0.4062	0.3774	0.3809	0.4062	0.3367	0.3508	0.3327	0.3957	0.4113	0.3890	0.3858	0.3207	0.4416	0.4350	0.3082	0.2383
[58]Northern-Thailand-Thailand-Shan	0.0187	0.0243	-0.0054	0.0397	0.0337	0.0148	0.0890	0.0315	0.0456	0.0168	0.0403	0.0418	0.0420	0.0158	0.1053	0.0486	0.0571	0.0799	0.0303	0.0411	0.0223	0.0448	0.1111	0.0902	0.0798	0.0961	0.1741	0.1141	0.0078	-0.0244
[59]Central-Thailand-Thailand-Thai	0.0190	0.0309	0.0112	0.0592	0.0414	0.0192	0.0786	0.0320	0.0454	0.0191	0.0376	0.0391	0.0278	0.0218	0.0942	0.0452	0.0686	0.0969	0.0345	0.0399	0.0338	0.0409	0.1142	0.0974	0.0563	0.0793	0.2037	0.1243	0.0301	0.0108
[60]Northern-Thailand-Thailand-Northern-Thai	0.0269	0.0079	0.0001	0.0614	0.0706	0.0289	0.1127	0.0475	0.0531	0.0392	0.0580	0.0602	0.0258	0.0311	0.1073	0.0474	0.0674	0.0916	0.0472	0.0546	0.0390	0.0757	0.1225	0.0994	0.0674	0.0589	0.1796	0.1218	0.0206	-0.0125
[61]Northern-Thailand-Thailand-Yong	0.0298	0.0592	0.0260	0.0571	0.0360	0.0403	0.0897	0.0500	0.0609	0.0369	0.0529	0.0562	0.0489	0.0290	0.1130	0.0640	0.0840	0.1170	0.0514	0.0568	0.0558	0.0511	0.1232	0.1110	0.0897	0.1035	0.2110	0.1456	0.0452	0.0116

Supplementary Table S10. The pairwise Rst genetic distances between the three studied populations and 58 Chinese reference populations (Asian populations).

Population	[31]	[32]	[33]	[34]	[35]	[36]	[37]	[38]	[39]	[40]	[41]	[42]	[43]	[44]	[45]	[46]	[47]	[48]	[49]	[50]	[51]	[52]	[53]	[54]	[55]	[56]	[57]	[58]	[59]	[60]	[61]
[32]Southern-India-India-Tamil	0.0528																														
[33]Andhra-Pradesh-India-Thoti	0.0498	-0.0274																													
[34]Gunma-Japan-Japanese	0.1848	0.1916	0.2211																												
[35]Shizuoka-Japan-Japanese	0.1856	0.1945	0.2199	-0.0002																											
[36]Tokyo-Japan-Japanese	0.1817	0.1969	0.2354	-0.0038	-0.0057																										
[37]Tsukuba-Japan-Japanese	0.1533	0.1643	0.1913	0.0038	-0.0028	-0.0029																									
[38]Daejeon-South-Korea-Korean	0.1920	0.1805	0.2425	0.0640	0.0396	0.0424	0.0225																								
[39]Seoul-South-Korea-Korean	0.1829	0.1708	0.2269	0.0771	0.0570	0.0599	0.0363	-0.0015																							
[40]Ulaanbaatar-Mongolia-Mongolian	0.1864	0.1015	0.0452	0.2844	0.2679	0.2890	0.2539	0.2599	0.2549																						
[41]Singapore-Han	0.1274	0.1380	0.2076	0.1263	0.1137	0.1051	0.0809	0.0684	0.0658	0.2241																					
[42]Singapore-Indian	0.0422	0.0088	0.0061	0.1831	0.1809	0.1826	0.1524	0.1620	0.1535	0.1355	0.1176																				
[43]Singapore-Malay	0.0841	0.0876	0.0726	0.0964	0.1116	0.1097	0.0895	0.1184	0.1249	0.1946	0.0531	0.0760																			
[44]Taiwan-Ami	0.2119	0.1875	0.2189	0.2753	0.2464	0.2624	0.2191	0.2639	0.2528	0.2028	0.1736	0.1740	0.0970																		
[45]Taiwan-Atayal	0.3782	0.3302	0.4606	0.4501	0.3985	0.4250	0.3573	0.4324	0.4084	0.3240	0.3866	0.3425	0.2350	0.2494																	
[46]Taiwan-Bunun	0.3440	0.3723	0.4068	0.3817	0.3528	0.3808	0.3301	0.4060	0.3938	0.3756	0.3505	0.3598	0.2547	0.2800	0.4066																
[47]Taiwan-Paiwan	0.3361	0.3117	0.3747	0.3726	0.3372	0.3650	0.3112	0.3671	0.3579	0.3019	0.2969	0.3060	0.1809	0.1779	0.2374	0.2131															
[48]Taiwan-Puyuma	0.2304	0.2089	0.1973	0.2667	0.2487	0.2675	0.2272	0.2740	0.2751	0.2577	0.1991	0.2071	0.0855	0.1399	0.1343	0.2391	0.0522														
[49]Taiwan-Rukai	0.3472	0.3055	0.3520	0.3908	0.3571	0.3827	0.3307	0.3852	0.3770	0.2904	0.3132	0.3053	0.1916	0.1737	0.1303	0.2614	0.0252	0.0234													
[50]Taiwan-Saisiat	0.2298	0.2284	0.2597	0.2644	0.2278	0.2522	0.2018	0.2276	0.2262	0.2355	0.1516	0.2325	0.1190	0.1822	0.3107	0.3013	0.1948	0.0708	0.1835												
[51]Taiwan-Tsou	0.4574	0.4135	0.5930	0.5159	0.4688	0.4996	0.4215	0.4935	0.4655	0.3834	0.4638	0.4206	0.3098	0.3641	0.3084	0.3703	0.2250	0.2520	0.1859	0.4297											
[52]Taiwan-Yami	0.3429	0.3075	0.4192	0.3764	0.3228	0.3470	0.2948	0.3430	0.3380	0.2827	0.2931	0.3167	0.1983	0.2585	0.2202	0.4269	0.2289	0.0835	0.1712	0.1052	0.4576										
[53]Northern-Thailand-Thailand-Karen-Padong	0.1972	0.2925	0.4007	0.2130	0.1925	0.1559	0.1598	0.1937	0.1922	0.3939	0.1353	0.2537	0.1777	0.3702	0.6488	0.4988	0.5310	0.4205	0.5583	0.3938	0.7420	0.5653									
[54]Northern-Thailand-Thailand-Karen-Pwo	0.1612	0.1951	0.2488	0.1699	0.1738	0.1521	0.1415	0.1490	0.1467	0.2812	0.0667	0.1679	0.0717	0.2554	0.5238	0.4352	0.4010	0.2735	0.4120	0.2523	0.6281	0.4189	0.0929	-							
[55]Northern-Thailand-Thailand-Karen-Skaw	0.1377	0.1724	0.2057	0.1246	0.1352	0.1131	0.1083	0.1137	0.1165	0.2823	0.0517	0.1440	0.0539	0.2320	0.4393	0.3973	0.3521	0.2204	0.3595	0.2124	0.5374	0.3406	0.0843	-0.0356							
[56]Northern-Thailand-Thailand-Lawa	0.2469	0.3137	0.5216	0.2583	0.2293	0.1831	0.1933	0.2536	0.2532	0.4119	0.2122	0.2752	0.1807	0.4090	0.6699	0.5639	0.5995	0.4764	0.6027	0.4935	0.8038	0.6374	0.1072	0.1709	0.1281						
[57]Northern-Thailand-Thailand-Lua	0.3976	0.4372	0.7087	0.4340	0.3838	0.3464	0.3146	0.3695	0.3491	0.5171	0.3837	0.4038	0.3390	0.5403	0.7740	0.6736	0.7292	0.6673	0.7370	0.6584	0.8588	0.7706	0.2974	0.4158	0.3409	0.3928					
[58]Northern-Thailand-Thailand-Shan	0.0903	0.1298	0.1755	0.0792	0.0743	0.0547	0.0476	0.0745	0.0785	0.2384	0.0188	0.1017	0.0167	0.1835	0.4835	0.3775	0.3731	0.2314	0.3867	0.2163	0.6165	0.3900	0.0449	-0.0003	-0.0143	0.0980	0.3448				
[59]Central-Thailand-Thailand-Thai	0.0993	0.1264	0.1701	0.0942	0.1015	0.0832	0.0711	0.0793	0.0811	0.2397	0.0225	0.1075	0.0298	0.1520	0.3347	0.3114	0.2773	0.1947	0.2998	0.1785	0.4085	0.2891	0.0848	0.0391	0.0259	0.1209	0.2475	-0.0210			
[60]Northern-Thailand-Thailand-Northern-Thai	0.0786	0.1292	0.1744	0.0840	0.0778	0.0545	0.0470	0.0782	0.0798	0.2510	0.0524	0.1041	0.0329	0.2118	0.4852	0.3862	0.3845	0.2496	0.4087	0.2489	0.6023	0.3972	0.0491	0.0420	0.0200	0.1009	0.2738	-0.0502	-0.0132		
[61]Northern-Thailand-Thailand-Yong	0.1429	0.1646	0.2333	0.1222	0.1098	0.0898	0.0756	0.0896	0.0906	0.2524	0.0367	0.1416	0.0521	0.1737	0.3815	0.3562	0.3175	0.2308	0.3387	0.2086	0.4701	0.3152	0.1112	0.0619	0.0535	0.1244	0.2754	-0.0140	0.0114	-0.0062	

Supplementary Table S11. The Rst values between our studied populations and neighboring Asian Meta-populations.

Population	Zunyi-Han	Qiandongnan-Miao	Qiannan-Bouyei	China	Japan	Mongolia	Philippines	Taiwan	Thailand	Vietnam
Zunyi-Han										
Qiandongnan-Miao	0.0429									
Qiannan-Bouyei	0.0153	0.0171								
China	0.0025	0.0601	0.0259							
Japan	0.0809	0.1055	0.0862	0.0846						
Mongolia	0.1718	0.2587	0.2252	0.1312	0.2649					
Philippines	0.0572	0.1513	0.1074	0.0462	0.1457	0.1493				
Taiwan	0.2257	0.3375	0.2888	0.1703	0.2920	0.2767	0.1117			
Thailand	0.0382	0.0415	0.0198	0.0529	0.0935	0.2719	0.1077	0.2685		
Vietnam	0.0020	0.0200	0.0016	0.0163	0.0727	0.2074	0.0767	0.2713	0.0078	
